# Dissociable multi-scale patterns of development in personalized brain networks

**DOI:** 10.1038/s41467-022-30244-4

**Published:** 2022-05-12

**Authors:** Adam R. Pines, Bart Larsen, Zaixu Cui, Valerie J. Sydnor, Maxwell A. Bertolero, Azeez Adebimpe, Aaron F. Alexander-Bloch, Christos Davatzikos, Damien A. Fair, Ruben C. Gur, Raquel E. Gur, Hongming Li, Michael P. Milham, Tyler M. Moore, Kristin Murtha, Linden Parkes, Sharon L. Thompson-Schill, Sheila Shanmugan, Russell T. Shinohara, Sarah M. Weinstein, Danielle S. Bassett, Yong Fan, Theodore D. Satterthwaite

**Affiliations:** 1 The Penn Lifespan Informatics and Neuroimaging Center, University of Pennsylvania, Philadelphia, PA 19104 USA; 2 Department of Psychiatry, Neurodevelopment & Psychosis Section, University of Pennsylvania, Philadelphia, PA 19104 USA; 3 Chinese Institute for Brain Research, 102206 Beijing, China; 4 Department of Radiology, the University of Pennsylvania, Philadelphia, PA 19104 USA; 5 Department of Pediatrics, College of Education and Human Development, University of Minnesota, Minneapolis, MN 55455 USA; 6 Department of Neurology, University of Pennsylvania, Philadelphia, PA 19104 USA; 7 Center for Biomedical Imaging and Neuromodulation, Nathan S. Kline Institute for Psychiatric Research, Orangeburg, NY 10962 USA; 8 Center for the Developing Brain, Child Mind Institute, New York City, NY USA; 9 Department of Bioengineering, University of Pennsylvania, Philadelphia, PA 19104 USA; 10 Department of Psychology, University of Pennsylvania, Philadelphia, PA 19104 USA; 11 Department of Biostatistics, Epidemiology, and Informatics, University of Pennsylvania, Philadelphia, PA 19104 USA; 12 Department of Electrical & Systems Engineering, University of Pennsylvania, Philadelphia, PA 19104 USA; 13 Department of Physics & Astronomy, University of Pennsylvania, Philadelphia, PA 19104 USA; 14 Santa Fe Institute, Santa Fe, NM 87051 USA

**Keywords:** Neuronal development, Intelligence

## Abstract

The brain is organized into networks at multiple resolutions, or scales, yet studies of functional network development typically focus on a single scale. Here, we derive personalized functional networks across 29 scales in a large sample of youths (n = 693, ages 8–23 years) to identify multi-scale patterns of network re-organization related to neurocognitive development. We found that developmental shifts in inter-network coupling reflect and strengthen a functional hierarchy of cortical organization. Furthermore, we observed that scale-dependent effects were present in lower-order, unimodal networks, but not higher-order, transmodal networks. Finally, we found that network maturation had clear behavioral relevance: the development of coupling in unimodal and transmodal networks are dissociably related to the emergence of executive function. These results suggest that the development of functional brain networks align with and refine a hierarchy linked to cognition.

## Abstract (web summary)

Studies of brain network development typically focus on a single scale. Here, the authors derived personalized functional networks across scales, and find that network development systematically adheres to and strengthens hierarchical cortical organization.

## Introduction

Graded transitions from bottom-up, feedforward projections to top-down, feedback projections create an anatomic hierarchy of both regional^1,2^ and global^3,4^ cortical organization. In turn, anatomical hierarchy supports a hierarchy of cortical function. Whereas regional hierarchical organization facilitates higher-order stimulus encoding in sensory networks^1^, global hierarchical organization is thought to facilitate the development of executive functioning (EF)^5–7^. Critically, initial evidence suggests that global hierarchical organization is not established in youth, but instead is a product of protracted development^8–10^. Understanding the normative process by which hierarchical cortical organization emerges and supports EF is crucial, as deficits in the emergence of EF are associated with lower academic achievement^11,12^, risk-taking behaviors^13^, and most major psychiatric illnesses^14–16^.

Large-scale patterns of functional organization can be identified in humans using functional MRI (fMRI), which allows for studies of development and cognition. Prior developmental neuroimaging studies have found that a sensorimotor to association hierarchy represents a principal mode of functional coupling in adults^17^, but not in infants^8^ or children^9,10^. These results implicate development as central in the establishment of a normative cortical hierarchy, but the process by which this hierarchy emerges is unclear. In parallel, recent studies of cognition in neurodevelopment have found that functional segregation of cortical networks near the top of the hierarchy from lower-order networks supports the emergence of EF^18–20^. Although these results further suggest a role of functional hierarchy in cognitive development, other studies have produced discrepant results^21–24^, leaving the role of cortical hierarchy in cognitive development unclear. This lack of consensus across existing work may arise due to two limitations that are shared across prior studies.

First, nearly all studies of functional network development only examine a single network resolution or scale. Typically, investigators use standard network atlases that specify a single number of functional networks (e.g., 7, 14, or 17). However, it is increasingly recognized that the brain is a multi-scale system, and that studies of a specific resolution of subnetworks may be limited^25–28^. Rather, evidence suggests that brain network organization emerges from neural coordination across overlapping spatial scales^25,29–31^. Importantly, distinct brain-behavior relationships may be present at different scales^32^, with each scale potentially offering complementary information regarding multifaceted processes such as development. As a result, current accounts of brain development that rely on a single network scale are almost certainly incomplete and may hamper our ability to synthesize findings across studies where different scales were examined^33,34^.

A second key limitation of prior studies of functional network development is that they have not accounted for individual differences in the spatial layout of brain networks on the cortical mantle. Multiple independent studies in adults using different datasets and distinct methods have provided convergent evidence that there is prominent between-individual variability in the spatial distribution (i.e., the functional topography) of large-scale networks on the anatomic cortex^35–39^. In studies of adults, transmodal association networks tend to have the greatest variability in functional topography^36–39^; recent work has shown that this is also true in children and adolescents^40^. Accounting for such individual variation in functional topography may be critical for understanding the development of coupling between networks, as prior work has shown that differences in spatial topography can be aliased into estimates of connectivity^35,41^. Further, individual differences in spatial topography and individual differences in connectivity can have distinct associations with psychopathology^42^. Finally, individual-specific–or “personalized”–networks may be particularly relevant when evaluating development at multiple scales, as individual variation in topography might depend in part on network resolution^43,44^.

In this study, we sought to understand how multi-scale cortical networks, occupying diverse positions across the sensorimotor-association hierarchy, mature with age to support EF. We evaluated the development of multi-scale personalized networks in a large sample of youth, with the goal of testing three interrelated hypotheses. First, we hypothesized that across scales, patterns of network development would vary across the sensorimotor-association hierarchy, with association networks exhibiting functional segregation relative to sensorimotor networks. Second, we predicted that association network segregation would relate to the maturation of EF in adolescence. Finally, we expected to find evidence of multi-scale network development. Specifically, given the diverse functions supported by brain organization at different scales, we anticipated that different network scales would have distinct associations with both age and EF.

## Results

**Fig. 1:**
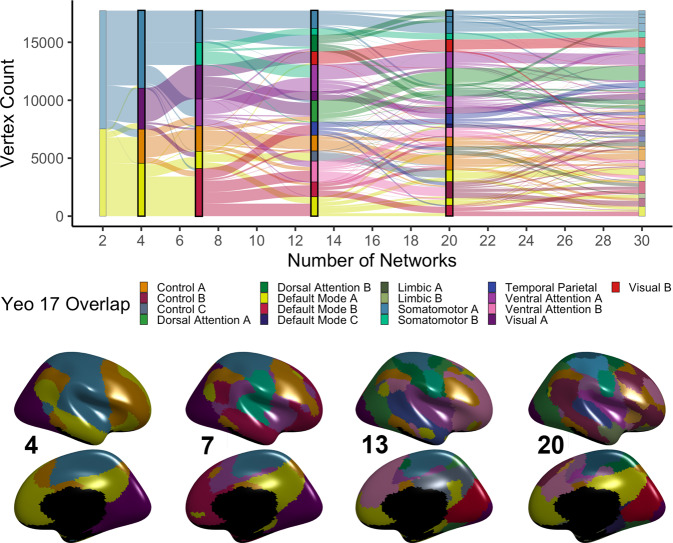
Group-consensus functional networks at multiple scales. We used regularized non-negative matrix factorization (see Supplementary Fig. 1) to derive personalized functional networks at 29 scales (2–30 networks). Tracking network membership of each vertex across scales reveals a nested structure where finer-grained networks gradually emerge from coarse networks (top). Scales 4, 7, 13, and 20 are chosen for visualization; see bottom panel for cortical projections. Colors reflect each network’s predominant overlap with a canonical atlas of 17 functional networks^84^.

We studied 693 youths ages 8–23 years from the Philadelphia Neurodevelopmental Cohort, who completed fMRI at 3 T and had 27 min of high-quality data^41,45^. To derive multi-scale personalized functional networks, we used a specialized adaptation of non-negative matrix factorization (NMF) that incorporates spatial regularization^46,47^ (see Methods, Figure S1). To ensure correspondence of personalized networks across participants, this process was initialized by creating a group atlas, which was then adapted to each individual’s data (see Methods). To evaluate multiple resolutions, group atlases that included between 2 and 30 networks were created (Fig. 1 and Fig. S2A). Across this range of scales, reconstruction error declined smoothly (Fig. S2B). To evaluate the degree to which finer-grained functional networks were nested within the network partitions obtained at the coarsest scale, we evaluated each network for its spatial overlap with the group atlas derived at K = 2 networks. Across scales, ~57% of all networks fell within the unimodal partition, and 43% fell within the transmodal partition (Fig. S3).

**Fig. 2:**
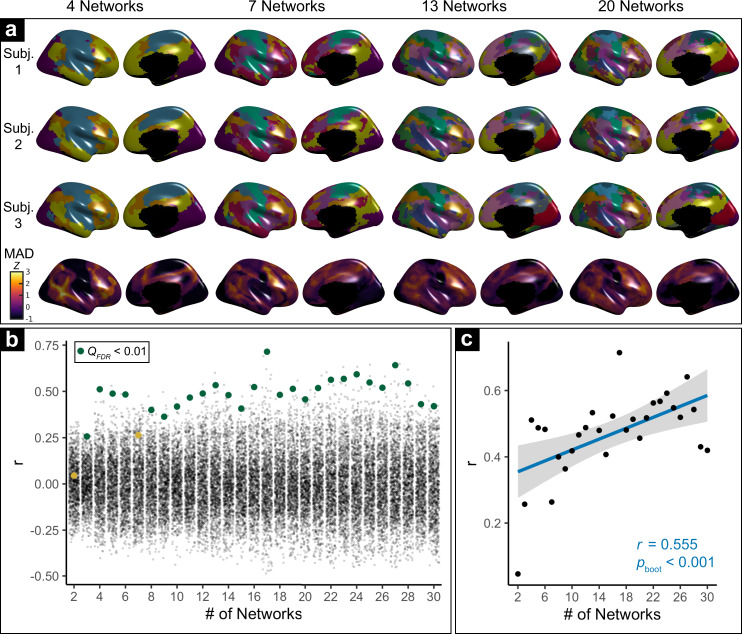
Variability in personalized networks across scales. **a** Variability in personalized networks is greatest in association cortex across scales. Exemplar personalized networks at scales 4, 7, 13, and 20 are shown for three participants. Prominent individual differences in functional topography are present at all scales, as quantified by median absolute deviation (MAD) of functional network loadings across participants (bottom row, z-scored within each scale). **b** Variability of functional topography aligns with functional hierarchy. Spin-tests of the correlation between topographic variability and the principal functional connectivity gradient^17^ at each scale reveal that variability is significantly correlated with a sensorimotor-to-association hierarchy at most scales (green dots = significant correlations; yellow dots = non-significant correlations; black dots = spin-test null correlations, FDR false discovery rate). **c** Greater alignment between a sensorimotor-to-association hierarchy and topographic variability is present at finer scales. Scatterplot depicts second-order correlation of variability (MAD) and the principal gradient (from **b**) across scales. The statistical test is two-sided. Error bands depict the 95% confidence interval.

Examination of multi-scale personalized brain networks revealed prominent differences in person-specific functional neuroanatomy at all scales (Fig. 2a and Fig. S4); networks were robust to NMF parameters chosen (Fig. S5). Prior work at a single scale found that variability in functional neuroanatomy disproportionately localizes to association cortices^35–39^. Here, to quantify individual variability in-network topography, we calculated the median absolute deviation (MAD) of network loadings at each cortical vertex across participants. To verify that variability was consistently greater within association cortices at multiple scales, we compared network MAD at each scale to a widely used map summarizing a functional hierarchy, derived from the principal gradient of functional connectivity^17^ (see Methods). Using a conservative spin-based spatial randomization procedure that accounts for spatial auto-correlation^48^, we found that MAD was positively correlated with functional hierarchy in 27 of the 29 scales evaluated (Fig. 2b; green). Furthermore, we found that topographic variability became increasingly correlated with the hierarchy at finer scales (Fig. 2c; *r* = 0.56, *p*_boot_ < 0.001). These results demonstrate that variability in functional neuroanatomy is increasingly prominent within association cortices at finer-grained network resolutions.

### Brain network coupling develops according to a hierarchical sensorimotor-association axis

**Fig. 3:**
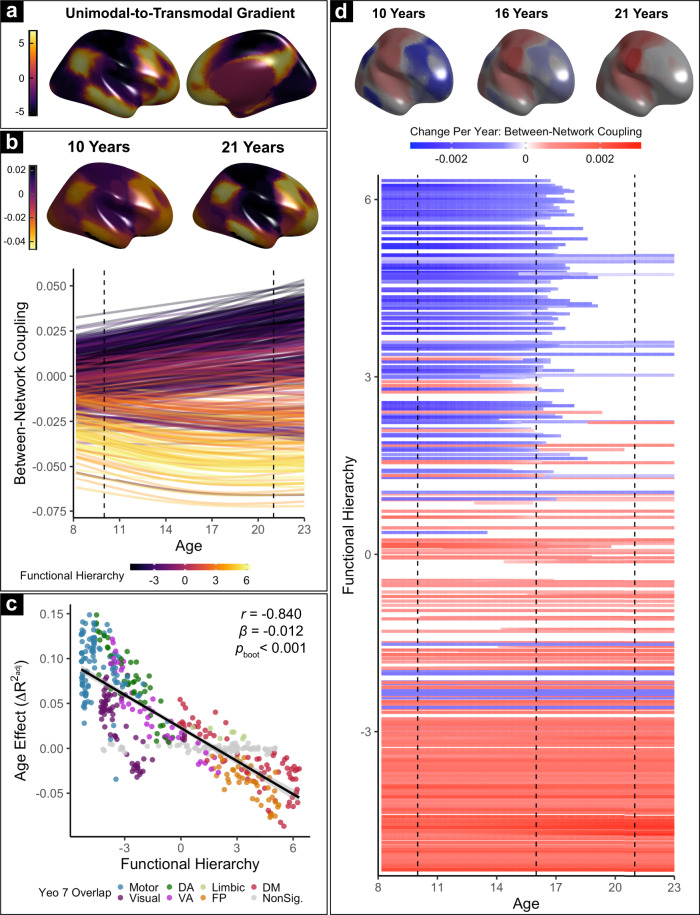
Network development in youth unfolds along a functional hierarchy. **a** We define functional hierarchy according to the widely used principal gradient of functional connectivity from Margulies et al. (2016), which describes each location on the cortex on a unimodal-to-transmodal continuum. **b** Between-network coupling is modeled for every network at each scale using Generalized Additive Models (GAMs) with penalized splines to account for linear and nonlinear effects of age. Each solid line represents the developmental pattern of one network at one scale; colors indicate the position of that network on the functional hierarchy. Dashed lines and corresponding brain maps represent estimated between-network coupling at each age, averaged across scales. Between-network coupling of sensorimotor networks (purple lines) increases with age, indicating increased integration. In contrast, the coupling of association networks (yellow lines) declines with age, reflecting increased segregation. **c** Age effects of each network (from **b**) are plotted versus their position on the functional hierarchy (from **a**). Networks that do not display significant change over development are shaded in gray (*Q*_FDR_ > 0.05). The position of each network on the functional hierarchy explains the majority of variance in age effects (*r* = −0.840, *β* = −0.012, *p*_boot_ < 0.001, two-sided). **d** We quantified the duration, magnitude, and direction of maturational changes in coupling for each network using the derivatives of the fitted splines (from **b**). Top: annualized change in between-network coupling at 10, 16, and 21 years old, averaged across scales. Bottom: change per year in average between-network coupling of each network across the age range studied; as in **b**, each line represents the developmental pattern of a given network at a single scale. While integration of sensorimotor networks increases over the entire age range sampled, segregation of association networks generally plateaus near the end of adolescence.

Having defined multi-scale personalized networks in a large sample of youth, we next sought to examine how network coupling evolves with age. To summarize the functional coupling of each network to other networks, we averaged between-network connectivity values across all personalized networks at each scale (Fig. S6). We hypothesized that age-related changes in between-network coupling would vary according to a network’s position on the sensorimotor-association functional hierarchy. To test this hypothesis, we first summarized each networks’ position along the functional hierarchy, where higher values correspond to regions located in association cortices and lower values are assigned to regions in sensorimotor cortices (Fig. 3a). Specifically, the position of each network in the functional hierarchy was operationalized by extracting the average value of the principal gradient of functional connectivity^17^ within each network’s boundaries. We related all network-level age effects to this measure of functional hierarchy.

Across all participants and independent of age, we found greater average between-network coupling was present lower in the functional hierarchy, whereas attenuated coupling was present higher in the hierarchy (Fig. 3b). To rigorously model linear and nonlinear changes in coupling over development, we used generalized additive models (GAMs) with penalized splines to examine how between-network coupling of each network was associated with age. In these models, sex and in-scanner motion were also included as covariates. We found that age-related changes in between-network coupling were largely explained by a network’s position in the functional hierarchy. Between-network coupling of lower-order networks became more positive at older ages, indicative of greater network integration. In contrast, between-network coupling in higher-order networks became more negative, reflecting increasing segregation. A network’s position on the functional hierarchy explained most of the variance in observed developmental effects (Fig. 3c; *r* = −0.84, *p*_boot_ < 0.001). Sensitivity analyses yielded similar results using data from resting-state scans alone (Fig. S7a, b, d; *r* = −0.77, *p*_boot_ < 0.001) or from task scans alone (Fig. S8a, b, d; *r* = −0.83, *p*_boot_ < 0.001). Together, these results suggest that the development of between-network coupling in youth is largely described by dissociable processes of segregation and integration across the functional hierarchy.

Next, we sought to identify intervals of significant age-related change in-network coupling. To accomplish this, we calculated the confidence interval of the derivative of the developmental curve for each model. We found that age-related changes in sensorimotor and association networks occurred over different developmental periods: between-network coupling increased in lower-order areas over the entire age range studied, whereas decreases in between-network coupling in higher-order areas did not extend beyond late adolescence (Fig. 3d). Consequently, in addition to differences in the sign of developmental changes described above, the temporal span of maturation in-network coupling also systematically varied across the cortico-functional hierarchy.

**Fig. 4:**
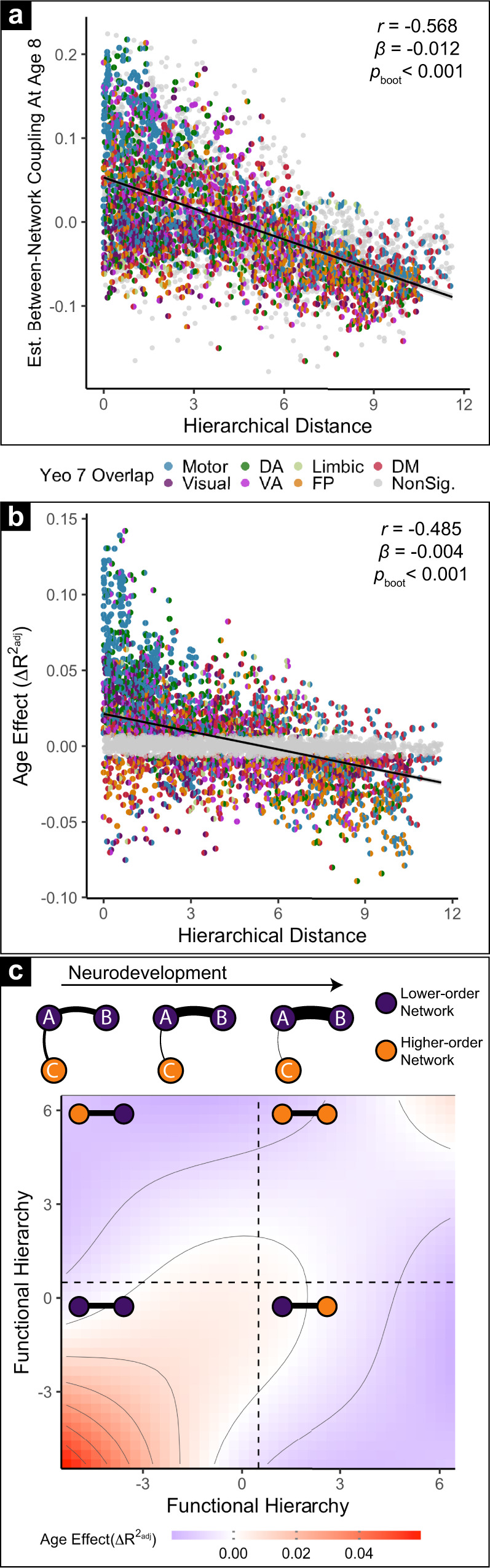
Maturation of between-network coupling aligns with the position of each network in the functional hierarchy. **a** Mean between-network coupling is largely captured by relative position along the sensorimotor to association axis. The inter-network coupling of each pair of networks at each scale is modeled using a GAM to estimate their values at age 8. Here, those values are plotted versus the difference in the hierarchical position of the two networks being evaluated. Each data point represents the coupling of a network pair at a given scale. Each half of the circle is colored according to constituent networks’ maximum overlap with the 7-network solution defined by Yeo et al. (2011); network pairs that do not significantly change with age after FDR correction (*Q* < 0.05) are shaded in gray. As expected, networks at a similar position along the functional hierarchy tend to have higher coupling (*r* = −0.568, *β* = −0.012, *p*_boot_ < 0.001, two-sided). **b** Age effects quantifying the development of between-network coupling is similarly aligned with the relative position of networks along the functional hierarchy. Age effects of every network pair at each scale are plotted versus their hierarchical distance and colored as in **a**. Network pairs without significant age effects are plotted in gray. Developmental effects on pairwise coupling between networks are associated with the hierarchical distance between networks (*r* = −0.49, *p*_boot_ < 0.001, two-sided). **c** Top: schematic summarizing developmental effects. Development is associated with strengthening of network coupling between lower-order networks and weakening of coupling between lower and higher-order networks; thicker lines represent greater functional coupling. Bottom: topographical plot of the observed age effect as a function of absolute (rather than relative) network hierarchy values across all network pairs. Increased coupling with age between functionally similar networks is prominent for sensorimotor networks (bottom left), and less prominent for association networks (top right). Age-related decreases in coupling occur in sensorimotor-association network pairs (top left and bottom right).

To provide a more nuanced understanding of the maturational changes in between-network coupling described above, we next evaluated the development of specific connections between networks. As between-network connections can link networks that have a similar hierarchical position (i.e., two association networks) or may alternatively link a sensorimotor and association network, we calculated the difference in hierarchical position of the two networks connected by each edge. As the principal axis captures variance in the cortical coupling, we expected networks similarly positioned along this axis to share a degree of this variance. As expected, we found that networks with similar hierarchical positions had greater mean coupling, and networks that were further apart in the functional hierarchy tended to have weaker coupling across participants (*r* = −0.57, *p*_boot_ < 0.001; Fig. 4a). Critically, we additionally found that age-related changes in-network edges were also explained by differences in their relative position in the functional hierarchy (*r* = −0.49, *p*_boot_ < 0.001; Fig. 4b). Specifically, sensorimotor-to-sensorimotor edges tended to strengthen with age, whereas edges that linked sensorimotor and association networks weakened (Fig. 4c; *p*_boot_ < 0.001); developmental strengthening of association-to-association edges was present but less prominent. Sensitivity analyses provided convergent results using data from resting-state scans only (Fig. S7c; *r* = −0.39, *p*_boot_ = 0.005) and from task scans only (Fig. S8c; *r* = −0.45, *p*_boot_ < 0.001). These results demonstrate that functional network development is characterized by increases in coupling between hierarchically similar networks and decreases in coupling between dissimilar networks—yielding increased differentiation along the functional hierarchy with development.

It should be noted that previous studies have documented that the physical distance between two brain regions explains the patterning of functional maturation across network edges^49–51^. As functional hierarchy is related to the intrinsic geometry of the cortex^52,53^, we sought to verify that the effects of hierarchical distance described above were not better explained by physical distance. To do so, we compared the correlation between age effects and Euclidean distance with the relationship between age effects and hierarchical distance. While the correlation between Euclidean distance and age effects was significant (*r* = −0.11, *p*_boot_ < 0.001; Fig. S9), it was substantially weaker than that observed for hierarchical distance (*r* = −0.49, *p*_boot_ < 0.001) and the effect of hierarchical distance remained significant while co-varying for Euclidean distance (partial *r* = −0.45, *p* < 0.001). This result suggests that although the physical distance spanned by a functional connection is weakly related to its developmental pattern, developmental effects are better explained by the functional distance that a connection spans across the sensorimotor-to-association hierarchy.

### Development has dissociable signatures at different networks and scales

**Fig. 5:**
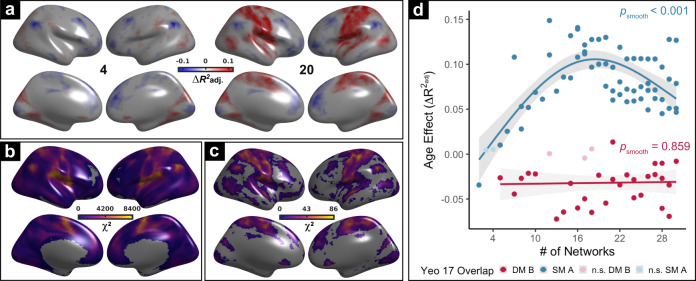
The interactions between-network scale and developmental coupling is maximal in sensorimotor cortex. **a** The effect of age on average vertex-wise between-network coupling at two scales (4 and 20). Age effects are modeled using GAMs with penalized splines; thresholded at *Q*_FDR_ < 0.05. Scale-dependent age effects can be observed in sensorimotor cortex: while no developmental increase in between-network coupling was seen in somatomotor cortex at scale 4, such an increase is evident at scale 20. **b** Across ages, between-network coupling of the sensorimotor cortex is strongly influenced by scale. Generalized estimating equations (GEEs) reveal that the effect of scale (*χ*^2^) differentially influences the strength of between-network coupling across the cortex. Locations within unimodal sensorimotor cortex exhibit the strongest scale-dependence in their mean between-network coupling (*Q*_FDR_ < 0.05). **c** Scale differentially interacts with age-dependent developmental associations with coupling across the cortex. GEEs are used to examine the degree of scale-moderated developmental effects (age-by-scale interaction; thresholded at *Q* < 0.05); maximal effects are present in the sensorimotor cortex. **d** Scale differentially interacts with age-dependent developmental effects in sensorimotor and association networks. Specifically, age effects in lower-order networks tend to be more scale-dependent than those in higher-order networks. The effect of age across scales is plotted for networks predominantly overlapping with the lowest-order (blue; Somatomotor-A) and highest-order (red; Default Mode-B) networks, as quantified from the functional hierarchy. Statistical tests are two-sided. Error bands depict the 95% confidence interval.

The above results demonstrate that functional network development is largely captured by a network’s position on a hierarchical axis of sensorimotor-to-associative function. However, these analyses are agnostic to the multi-scale nature of the personalized brain networks that we constructed. As a next step, we evaluated whether developmental effects were dependent on network scale. Initial inspection revealed that the relationship between age and between-network coupling varied systematically as a function of scale, with greater age effects in the sensorimotor cortex at finer network scales (Fig. 5a). To quantify scale effects while controlling for within-subject correlations over scales, we used generalized estimating equations (GEEs) with exchangeable correlation structures at each cortical vertex. We found that the effect of scale on between-network coupling was strongest in the sensorimotor cortex (Fig. 5b). Furthermore, we found evidence that scale-moderated age effects, with maximal scale-by-age interactions being observed in the sensorimotor cortex (Fig. 5c).

To further understand these scale-dependent age effects, we compared the age effect across scales for networks that fall at opposite ends of the sensorimotor-to-association hierarchy. Specifically, at each scale we identified networks that aligned most closely with the somatomotor-A network and the default mode-B network from the commonly used atlas defined by Yeo et al. (Fig. 5d). This comparison revealed that age effects within the somatomotor network were highly scale-dependent, with greater increases in between-network coupling with age at finer scales. In contrast, default-mode networks demonstrated consistent developmental segregation across scales. These results suggest that age-related changes in-network coupling are differentially linked to scale across the cortical hierarchy.

### Multi-scale network coupling is associated with executive function

**Fig. 6:**
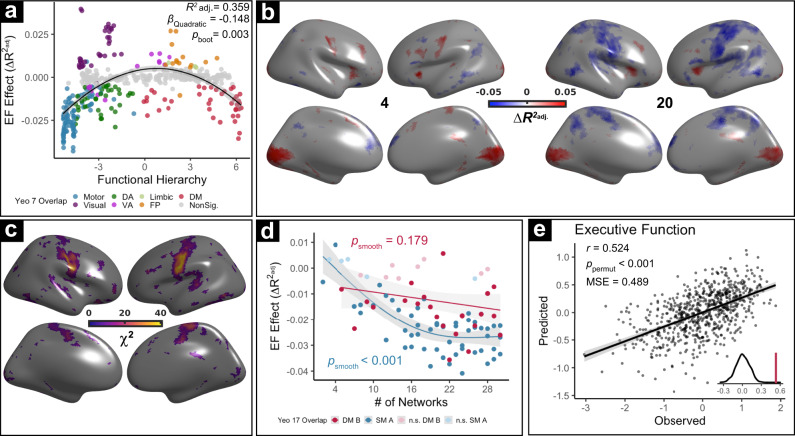
Multi-scale network coupling is associated with executive function. **a** Network-level relationships between coupling and EF are quadratically related to transmodality. Specifically, segregation of both sensorimotor and default-mode networks is associated with better EF. These associations with EF are dissociable from normative developmental effects (Fig. 3c) where default-mode segregation and sensorimotor integration are observed. The statistical test was two-sided. **b** Analyses at scales 4 and 20 reveal differing associations with EF. While between-network coupling of visual, insular, and dorsolateral prefrontal cortical areas is consistently associated with greater EF (*Q*_FDR_ < 0.05), opposite associations with EF were present in motor cortex at coarse and fine scales. **c** Tests of age-by-scale interactions using GEEs reveal that scale effects are strongest in the sensorimotor cortex. **d** Scale is differentially linked to EF associations with coupling in higher-order and lower-order networks. As for age, effects in somatomotor networks tend to be more scale-dependent than those in association networks. The effect of age across scales is plotted for networks predominantly overlapping with the lowest-order (blue; Somatomotor-A) and highest-order (red; Default Mode-B) of the Yeo 17 networks. **e** Complex patterns of multi-scale coupling between personalized networks accurately predicts EF in unseen data. Cross-validated ridge regression with nested parameter tuning was used to predict EF of unseen data using each participant’s multivariate pattern of coupling across scales. Error bands depict the 95% confidence interval, statistical tests are two-sided for **d** and one-sided for **e**. MSE = mean squared error.

Having delineated developmental changes in between-network coupling, we next sought to understand the implications for individual differences in executive function (EF). First, we modeled the association between-network coupling and EF, controlling for developmental effects by including age as a penalized spline; other model covariates included sex and motion as in prior analyses. We found that the relationship between EF and between-network coupling was quadratically related to the functional hierarchy (Fig. 6a; *p*_boot_ = 0.003); this quadratic pattern was markedly different than the linear relationship between hierarchy and age effects (see Fig. 3c for comparison). Specifically, decreased between-network coupling at both extremes of the hierarchy was associated with greater EF, with maximal effects being seen in sensorimotor and default-mode networks. In contrast, greater coupling of several visual, ventral attention, and fronto-parietal networks were associated with greater EF.

To further understand these effects, we next performed high-resolution analyses at each cortical vertex to detail associations between EF and between-network coupling across scales. Consistent with network-level results, reduced between-network coupling in default-mode regions like the medial prefrontal cortex and precuneus was associated with greater EF across scales (Fig. 6b). In contrast, greater between-network coupling in the dorsolateral prefrontal cortex, anterior insula, and calcarine fissure was associated with greater EF across scales. Sensorimotor cortices again exhibited scale-dependent associations: higher between-network coupling in the sensorimotor cortex was associated with reduced EF, but only at finer scales. To further assess the role of network scale, we used GEEs to examine whether there was an interaction between EF and scale on between-network coupling at each cortical location. This analysis revealed prominent scale effects, primarily in sensorimotor cortices (Fig. 6c). To further illustrate the differential effects of network scale, we again contrasted networks that lie at opposite ends of the functional hierarchy (Fig. 6d). We found that network scale did not moderate the association between default-mode network coupling and EF; greater default-mode segregation was associated with better EF across scales. However, somatomotor network associations with EF were highly dependent on network scale.

Having found evidence of both scale-dependent and scale-independent associations between EF and network coupling, we next examined the degree to which these complex patterns of coupling could jointly predict individual differences in EF. To do so, we fit a multivariate ridge regression model to predict EF using data from all scales, while controlling for age and in-scanner motion. We found that this multivariate model accurately predicted the EF of unseen participants (see Methods; Fig. 6e; *r* = 0.52, *p*_permut_ < 0.001). Similar results were obtained in sensitivity analyses that considered data only from resting-state or task fMRI runs (Fig. S7e, *r*_rest_ = 0.34, *p*_permut_ < 0.001; Fig. S8e, *r*_task_ = 0.54, *p*_permut_ < 0.001). These results emphasize that EF is supported by multi-scale patterns of functional coupling.

Finally, to assess the specificity of the relationship between functional network coupling and EF, we also evaluated associations with other major domains of cognition, including episodic memory and social cognition. For episodic memory, segregation of the most unimodal networks was similarly associated with episodic memory (Fig. S10a). However, transmodal segregation was not associated with episodic memory performance, and no quadratic relationship with functional hierarchy was observed (*p*_boot_ = 0.269). A similar assessment of the social cognition factor revealed no significant associations with network-level coupling after correction for multiple comparisons (Fig. S10b). Edge-level ridge regression analyses revealed reduced model performance for both episodic memory (*r* = 0.33, *p*_permut_ < 0.001, Fig. S10c) and social cognition (*r* = 0.14, *p*_permut_ = 0.024, Fig. S10d). Taken together, these results suggest some degree of specificity for links between multi-scale network connectivity and EF.

## Discussion

In this study, we demonstrated that variation in the development of person-specific functional networks is intrinsically related to fundamental properties of brain organization. Specifically, we found that developmental patterns differentially unfold along the hierarchical sensorimotor to association axis of organization: unimodal sensorimotor networks became more integrated with age, while transmodal association networks became more segregated. This dissociable pattern of maturation had unique relevance for the development of cognition: while greater segregation of association networks was associated with better EF, developmental integration of sensorimotor networks was associated with worse EF. By examining functional network development and associations with EF across a range of macroscale networks, we additionally identified scale-dependent effects, which were predominantly present in somatomotor networks. Taken together, these results provide a new framework that incorporates multi-scale cortical organization for understanding how functional network maturation allows for the development of cognition in youth.

### Functional network development differs by position in a unimodal to transmodal hierarchy

Previous work in adults^36–39^ has established that between-individual variability of functional topography is greatest in the association cortex. In our prior report^40^ we demonstrated that this is also true in youth. Such marked variability of functional topography in association cortices may be a result of protracted and environmentally sensitive development in these higher-order cortices, facilitating continuous adaptation to individual-specific needs^5,54^. Here, we extended prior findings by demonstrating that topographic variability aligns with a functional hierarchy across multiple network scales. Furthermore, we found that variability of functional topography increasingly localizes to association cortices as the number of functional networks increases. As this scale-dependency might be just one of many shifts in between-participant variability over scales^32,43^, our results highlight the importance of scale and precision functional mapping techniques for investigations of individual differences in functional network coupling.

We found strong evidence that developmental changes in between-network coupling align with a sensorimotor-to-association hierarchy. Even prior to adolescence, sensorimotor networks tended to have greater between-network coupling, which was primarily driven by their coupling with other lower-order networks. In contrast, association networks were more functionally segregated even among the youngest of our participants. From ages 8–23 years, this pattern became more prominent: between-network coupling further strengthened in lower-order networks and weakened with age in higher-order networks. Together, these developmental effects served to further distinguish the functional hierarchy that is now well described in adults and broadly aligns with recent reports using independent methods and datasets^9,10^. This functional differentiation of cortical hierarchy over development is consistent with evidence that cortical myeloarchitecture further differentiates between sensorimotor and association regions during adolescence^55^, and that higher-order structural networks become increasingly dissimilar from lower-order networks with age^56^. Coupling between hierarchically similar networks may be partially attributable to the propagation of infra-slow cortical waves along functional hierarchies^25,57–59^; however, additional research is needed to examine how such waves evolve in development. Taken together, our results suggest that functional network development in youth both aligns with and strengthens the sensorimotor-to-association hierarchy seen in adulthood.

### Functional network differentiation supports executive function

EF is supported by coordinated recruitment of distributed networks of brain regions^60–62^. We found that the segregation of networks located at the two opposing ends of the sensorimotor to association hierarchy (i.e., somatomotor and default-mode networks) was associated with cognitive performance. Conversely, we demonstrated that increased integration of networks more centrally positioned within the axis supported EF. As such, two dissociable patterns of normative network development observed across the cortical functional hierarchy differentially relate to the development of EF. Specifically, whereas normative developmental segregation of transmodal association networks was positively associated with EF, unimodal integration was positively associated with age but negatively associated with EF. These results in part explain the existing heterogeneous literature, which has reported that refinement of both functional network segregation and integration is important for neurocognitive development^19,63–65^. However, our results also specify that the degree to which developmental integration versus segregation is advantageous for EF may largely depend on a network’s role within the functional hierarchy.

That both sensorimotor and DMN segregation were associated with greater EF accords with recent work demonstrating that the overall balance of network activity shifts across the functional hierarchy when individuals are engaged in externally oriented versus internally guided cognition. Prior work has shown that localized activity within networks at the bottom of the hierarchy supports cognition when it is reliant on immediate perceptual input^66^. In contrast, greater segregation of unimodal networks from transmodal networks supports cognition that is dependent on internally-oriented processing, including memory or theory of mind^18,66,67^. Furthermore, the association between EF and integration of control networks situated more centrally in the hierarchy is supported by prior literature emphasizing the role of these networks in top-down control^68–70^. Speculatively, these results suggest that functional segregation at the extremes of the functional hierarchy, in tandem with the integration of control networks situated in the middle of the hierarchy, may serve to reduce cross-modal interference^71,72^ while facilitating coordination of brain networks specialized for top-down cognitive control^67–69^.

We found that transmodal cortical segregation increased with age in youth and is associated with enhanced EF. In contrast, unimodal cortical integration increased with age but was associated with poorer EF. This discrepancy could stem from differences in the pace of maturation between parts of the cortex. In late life, cortical networks reintegrate, losing the segregation that is achieved earlier in maturation^73–79^. Notably, this integration at the end of the lifespan has been shown to mediate cognitive decline in both normal aging and neurodegenerative disease^76–79^. Our data suggest that the inflection point between maturational segregation and integration may be temporally staggered across a normative hierarchy, with lower-order networks beginning reintegration prior to transmodal networks, which are still segregating in youth. Consequently, we hypothesize that processes seen in aging may have begun in lower-order sensorimotor networks in adolescence.

### Multi-scale patterns of network development are associated with executive function

Prior work has primarily investigated organizational regimes of 2^80^, 3^17^, 4^81^, 5^82^, 6^83^, 7^84^, 13^50^, and 17^84^ functional subdivisions of the brain. In line with an emergent body of literature regarding multi-scale brain organization^26,32,85,86^, the scale-dependencies that we observed suggest that previous, single-scale descriptions of neurodevelopment only partially describe cortical network reorganization in youth. Notably, we present new evidence that scale and hierarchical positioning interact. We observed differential effects of scale on both development and EF across the functional hierarchy, with scale effects being disproportionately present in unimodal cortices. Coarse segregation of unimodal networks from transmodal networks with age was concurrent with fine-grained integration within unimodal networks. In contrast, no such scale dependence was seen in transmodal networks. A similar scale-dependence was present in associations with EF: coarse segregation and fine-grained integration of motor areas were both associated with worse EF. These effects of network scale might be driven in part by a greater propensity for unimodal functional networks to host nested multi-scale organizations than their transmodal counterparts^87,88^.

Finer scales systematically capture shorter “neural bridges”^6^ across the functional hierarchy. In other words, as higher network resolutions distinguish increasingly similar subnetworks, finer scales ultimately capture functional interactions between networks that are more proximate in the functional hierarchy. In our data, at the coarsest scale of two functional subdivisions, between-network coupling reflects interactions between only a single sensorimotor and association network. At this resolution, network segregation between these two broad classes of cortex increased with age. In contrast, finer scales revealed that, along with overall developmental segregation of sensorimotor and association networks, there is prominent integration of functionally similar, finer-grained networks. Consequently, our findings illustrate that different network scales reveal different developmental effects across the functional hierarchy. Several limitations to the current study should be noted. Adolescent development represents a complex, layered process not easily delineated by cross-sectional studies. This is a particularly salient limitation for approaches seeking to establish the role of brain maturation in cognitive development, rather than their co-occurrence. Further, there are undoubtedly individual differences in the pace of brain development, which cannot be indexed with cross-sectional data^89^. Future longitudinal studies will be critical for understanding temporal precedence in network maturation and how deviations from normative neurodevelopment are associated with the emergence of psychopathology^90^. Second, as children tend to move more during MRI scans, in-scanner head motion continues to be a concern for all neuroimaging studies of development^91^. Here, we rigorously followed the best practices for mitigating the influence of head motion on our results, including the use of a top-performing preprocessing pipeline and co-varying for motion in all hypothesis testing^92^. The use of these conservative procedures limits the possibility that reported findings are attributable to in-scanner motion. Third, we used data combined across three fMRI runs, including two where an fMRI task was regressed from the data^93^. This choice was motivated by studies that have shown that functional networks are primarily defined by individual-specific rather than task-specific factors and that intrinsic networks during task performance are similarly organized to those at rest^94^. Importantly, by including task-regressed data, we were able to generate individualized networks with 27 min of high-quality data. Prior work suggests that parcellations created using a timeseries of this length show high concordance with those generated using 380 min of data^95^. Fourth, we studied multi-scale organization in the spatial domain; the brain also exhibits multi-scale organization in the temporal domain^96–99^. Future investigations using tools with greater temporal resolution may be critical for simultaneously describing the spatial and temporal multi-scale organization. Finally, the maturation of subcortical structures is a critical component of neurodevelopment^100,101^. Recent advances in precision^102,103^ and multi-scale^104^ functional mapping of subcortical regions and hierarchies^105^ present an excellent opportunity for future work to delineate the role of subcortical functional coupling in neurocognitive development.

In conclusion, we leveraged advances in delineating personalized functional networks to elucidate divergent patterns of functional network development and to establish their relevance for cognition. These results are important for understanding the developmental refinement of cortical hierarchy that is prominent in healthy adults. Moving forward, the process of this refinement may be critically important for understanding executive dysfunction in those affected by mental illness. Examining abnormalities of functional network reorganization in longitudinal clinical samples will provide an important opportunity to test the hypothesis that insufficient maturational segregation of association networks confers risk to diverse psychiatric disorders. Indeed, existing research suggests that abnormalities associated with cross-disorder psychopathology are predominantly present at the association end of the functional hierarchy^15,106,107^, and that diverse psychopathology is associated with attenuated segregation of higher-order networks^108^. Eventually, understanding the normative development of individualized networks may be a critical prerequisite for guiding personalized neuromodulatory interventions targeting both individual-specific functional neuroanatomy and developmental phases with amenable plasticity.

## Methods

### Participants

A total of 1601 participants were studied and compensated as part of the Philadelphia Neurodevelopmental Cohort^45^. We excluded 340 participants due to treatment with psychoactive medications, prior inpatient psychiatric treatment, or incidentally encountered structural brain abnormalities. Among the 1261 participants eligible for inclusion, 54 more were excluded from analyses due to low-quality T1-weighted images or low-quality FreeSurfer reconstructions. Of the 1207 subjects with useable T1 images and adequate FreeSurfer reconstructions, 514 more participants were excluded for missing functional data or poor functional image quality. For inclusion in analyses, all participants were required to have three functional runs that passed quality assurance. As prior^91,92^, a functional run was excluded if the mean relative root-mean square (RMS) framewise displacement was higher than 0.2 mm, or it had more than 20 frames with motion exceeding 0.25 mm. This set of exclusion criteria resulted in a final sample of 693 participants with a mean age of 15.93 years (*SD* = 2.33); the sample included 301 males and 392 females. All subjects or their parents/guardian provided informed consent, and minors provided assent. All study procedures were approved by the Institutional Review Boards of both the University of Pennsylvania and the Children’s Hospital of Philadelphia.

### Image acquisition

As previously described^45^, all MRI scans were acquired using the same 3 T Siemens Trim Trio whole-body scanner and 32-channel head coil and VB17 revision software at the Hospital of the University of Pennsylvania.

#### Structural MRI

Prior to functional MRI acquisitions, a 5 min magnetization-prepared, rapid acquisition gradient-echo T1-weighted (MPRAGE) image (TR = 1810 ms; TE = 3.51 ms; TI = 1100 ms, FOV = 180 × 240 mm^2^, matrix = 192 × 256, effective voxel resolution = 0.9 × 0.9 × 1 mm^3^) was acquired.

#### Functional MRI

We used one resting-state and two task-based (*n*-back and emotion identification) fMRI scans for the current study. All fMRI scans were acquired with the same single-shot, interleaved multi-slice, gradient-echo, echo-planar imaging (GE-EPI) sequence sensitive to BOLD contrast with the following parameters: TR = 3000 ms; TE = 32 ms; flip angle = 90°; FOV = 192 × 192 mm^2^, matrix = 64 × 64; 46 slices; slice thickness/gap = 3/0 mm, effective voxel resolution = 3.0 × 3.0 × 3.0 mm^3^. Resting-state scans consisted of 124 volumes, while the *n*-back and emotion recognition scans consisted of 231 and 210 volumes, respectively. Further details regarding the *n*-back^60^ and emotion recognition^109^ tasks have been described in prior publications.

#### Field map

A B0 field map was derived for application of distortion correction procedures, using a double-echo, gradient-recalled echo (GRE) sequence: TR = 1000 ms; TE1 = 2.69 ms; TE2 = 5.27 ms; 44 slices; slice thickness/gap = 4/0 mm; FOV = 240 mm; effective voxel resolution = 3.8 × 3.8 × 4 mm.

#### Scanning procedure

Before scanning, to acclimate subjects to the MRI environment, a mock scanning session where subjects practiced the task was conducted using a decommissioned MRI scanner and head coil. Mock scanning was accompanied by acoustic recordings of the noise produced by gradients coils for each scanning pulse sequence. During these sessions, feedback regarding head movement was provided using the MoTrack motion tracking system (Psychology Software Tools). Motion feedback was given only during the mock scanning session. To further minimize motion, before data acquisition, participants’ heads were stabilized in the head coil using a single foam pad over each ear and a third over the top of the head.

### Image processing

#### Preprocessing

Structural images were processed with FreeSurfer (version 5.3) to allow for the projection of functional timeseries to the cortical surface^110^. Functional images were processed using a top-performing preprocessing pipeline implemented using the eXtensible Connectivity Pipeline (XCP) Engine^111^, which includes tools from FSL^112,113^ and AFNI^114^. This pipeline included (1) correction for distortions induced by magnetic field inhomogeneity using FSL’s FUGUE utility, (2) removal of the initial volumes of each acquisition, (3) realignment of all volumes to a selected reference volume using FSL’s MCFLIRT, (4) interpolation of intensity outliers in each voxel’s timeseries using AFNI’s 3dDespike utility, (5) demeaning and removal of any linear or quadratic trends, and (6) co-registration of functional data to the high-resolution structural image using boundary-based registration^115^. Images were de-noised using a 36-parameter confound regression model that has been shown to minimize associations with motion artifacts while retaining signals of interest in distinct subnetworks^92^. This model included the six framewise estimates of motion, the mean signal extracted from eroded white matter and cerebrospinal fluid compartments, the mean signal extracted from the entire brain, the derivatives of each of these nine parameters, and quadratic terms of each of the nine parameters and their derivatives. Both the BOLD-weighted timeseries and the artifactual model timeseries were temporally filtered using a first-order Butterworth filter with a passband between 0.01 and 0.08 Hz to avoid mismatch in the temporal domain^116^. Furthermore, to derive timeseries that were more comparable across runs, the task activation model was regressed from *n*-back and emotion identification fMRI data^93^. The task activation model and nuisance matrix were regressed out using AFNI’s 3dTproject.

For each modality, the fMRI timeseries of each participant was projected to their own FreeSurfer surface reconstruction and smoothed on the surface of this reconstruction with a 6 mm full-width half-maximum kernel. The smoothed data were projected to the *fsaverage5* template, which has 10,242 vertices on each hemisphere (18,715 total vertices after removing the medial wall). Finally, we concatenated the three fMRI acquisitions, yielding a timeseries of 27 min and 45 s in total (555 volumes). As prior, we removed vertices with a low signal-to-noise ratio^117–119^. We used the same SNR mask as in our prior work, which used the same dataset^40^. After masking, 17,734 vertices remained for subsequent analyses.

#### Regularized non-negative matrix factorization

As previously described in detail^40,47^, we used non-negative matrix factorization^46,47^ (NMF) to derive personalized functional networks. The NMF method decomposes the timeseries by positively weighting cortical vertices that covary, leading to a highly specific and reproducible parts-based representation^46,120^. Our approach was enhanced by a group-consensus regularization term that preserves inter-individual correspondence, as well as a data locality regularization term to mitigate imaging noise, improve spatial smoothness, and enhance functional coherence of personalized functional networks (see Li et al., 2017 for details of the method; see also: https://github.com/hmlicas/Collaborative_Brain_Decomposition). As NMF requires non-negative input, we shifted the timeseries of each vertex linearly to ensure all values were positive. Finally, all vertex timeseries were normalized to their maximum values such that all values ranged between 0 and 1.

Given a group of *n* subjects, each having fMRI data X^*i*^ ∈ R^S × T^, *i* = 1, …, *n*, consisting of *S* vertices and *T* timepoints, we aimed to find *K* non-negative functional networks *V*^*i*^ = (*V*^*i*^_*s,k*_)∈*R*^*S* × *K*^ and their corresponding time courses *U*^*i*^ = (*U*^*i*^_*t,k*_)∈*R*^*T* × *K*^ for each subject, such that

1 $${X}^{i}\approx {U}^{i}\left({V}^{i}\right)^{\prime} +{E}^{i},s.t.{U}^{i},{V}^{i}\ge 0,\forall 1\le i\le n,$$

Where (*V*^*i*^)′ is the transpose of (*V*^*i*^) and *E*^*i*^ is independently and identically distributed residual noise following a gaussian distribution. Both *U*^*i*^ and *V*^*i*^ were constrained to be non-negative so that each functional network did not contain anticorrelated functional units. A group-consensus regularization term was applied to ensure inter-individual correspondence, which was implemented as a group-sparsity term on each column of *V*^*i*^ and formulated as

2 $${R}_{c}={\mathop{\sum }\limits_{k=1}^{K}{V}_{\cdot ,k}^{1,...,n}}_{2,1}=\mathop{\sum }\limits_{k=1}^{K}\frac{{\sum }_{s=1}^{S}({{\sum }_{i=1}^{n}({{V}_{s,k}^{i}})^{2}})^{1/2}}{({\sum }_{s=1}^{S}{\sum }_{i=1}^{n}{({{V}_{s,k}^{i}})^{2}})^{1/2}}$$

The data locality regularization term was applied to encourage spatial smoothness and coherence of the functional networks using graph regularization techniques^121^. The data locality regularization term was formulated as

3 $${R}_{M}^{i}={Tr}(({V}^{i})^{\prime} {L}_{M}^{i}{V}^{i}),$$

where ${L}_{M}^{i}={D}_{M}^{i}-{W}_{M}^{i}$ is a Laplacian matrix for subject *i*, ${W}_{M}^{i}$ is a pairwise affinity matrix to measure spatial closeness or functional similarity between different vertices, and ${D}_{M}^{i}$ is its corresponding degree matrix. The affinity between each pair of spatially connected vertices (here, vertices *a* and *b*) was calculated as $(1+corr({X}_{a}^{i},{X}_{b}^{i}))/2$, where *corr*$({X}_{a}^{i},{X}_{b}^{i})$ is the Pearson correlation coefficient between timeseries ${X}_{a}^{i}$ and ${X}_{b}^{i}$; the pairwise affinity between non-connected vertices was set to zero so that ${W}_{M}^{i}$ would be sparse. We identified personalized functional networks by optimizing a joint model with integrated data fitting and regularization terms formulated as

4 $$\mathop{\min }\limits_{\left({U}^{i},{V}^{i}\right)}\,\mathop{\sum }\limits_{i=1}^{n}\left({X}^{i}-{U}^{i}{\left({V}^{i} \right)}\right)_{F}^{2}+{\lambda }_{M}\mathop{\sum }\limits_{i=1}^{n}{R}_{m}^{i}+{\lambda }_{c}{R}_{c,}\\ s.t.{U}^{i}, {V}^{i}\ge 0,{V}_{\!.,k{{\infty }}}^{i}=1,\forall 1\le k\le K,\forall 1\le i\le n$$

Where ${\lambda }_{M}=\beta \times (T/K\times {n}_{m})$ and ${\lambda }_{c}=\alpha \cdot (n\cdot T/K)$ are used to balance the data fitting, data locality, and group-consensus regularization terms, *n*_*m*_ is the number of neighboring vertices, and *α* and *β* are free parameters leveraged to scale sparsity and locality in derived network solutions, respectively. For this study, we used previously validated parameters^40,47^ (Sparsity, locality = 1,10) across 29 values of *K* (*K* *=* 2 to *K* = 30) corresponding to 29 scales of cortical organization. To evaluate the spatial nesting of finer-grained functional networks within coarser networks, we evaluated the degree to which each network from K = 3 to K = 30 overlapped with the coarse network partitions derived at K = 2. Specifically, each vertex from the *fsaverage5* template was assigned to one of the two networks derived at K = 2, corresponding to a single unimodal and transmodal network. At subsequent (finer) scales, we evaluated A) which of the K = 2 networks that it predominantly overlapped within space (e.g., unimodal or transmodal) and B) the percentage of vertices that fell within that K = 2 network.

#### Defining personalized networks

Our approach to defining personalized networks included three steps. In the first two steps, a group-consensus atlas was created. In the third step, this group atlas was used to initialize network personalization for each participant at each scale. Although individuals exhibit distinct network topography, broad consistencies exist among individual-to-individual^39,94^. By first generating a group atlas for personalization initialization, we ensured spatial correspondence across all subjects and scales. This strategy has also been applied in other studies of personalized networks^121,122^. For computational efficiency and to avoid outlier-driven group atlases, a bootstrap strategy was utilized to perform the group-level decomposition multiple times on a subset of randomly selected participants. Subsequently, the resulting decompositions were fused to obtain one robust initialization. As prior^40,47^, we randomly selected 100 subjects and temporally concatenated their timeseries, resulting in a timeseries matrix with 55,500 rows (timepoints) and 17,734 columns (vertices). We applied the above-mentioned regularized non-negative matrix factorization method with a random initialization to decompose this group-level matrix^46^. A group-level network loading matrix *V* was acquired, which had *K* rows and 17,734 columns. Each row of this matrix represents a functional network, while each column represents the loadings of a given cortical vertex. As prior^40,46^, this procedure was repeated 50 times, each time with a different subset of subjects. Accordingly, this process yielded 50 different group atlas estimations for each value of *K*.

Next, we combined the 50 group network atlases to obtain one robust group network atlas with spectral clustering at each value of *K*. Specifically, we concatenated the 50 group parcellations together across networks to obtain a matrix with 50 × *K* rows (functional networks) and 17,734 columns (vertices). Next, we calculated inter-network similarity as

5 $${S}_{{ij}}={{\exp }}\left(-\frac{{d}_{{ij}}^{2}}{{\sigma }^{2}}\right),$$

where ${d}_{{ij}}=1-{{{{{{\mathrm{corr}}}}}}}\left({{{{{{{\mathrm{Network}}}}}}}}_{i},{{{{{{{\mathrm{Network}}}}}}}}_{j}\right),$ ${{{{{{\mathrm{corr}}}}}}}\left({{{{{{{\mathrm{Network}}}}}}}}_{i},{{{{{{{\mathrm{Network}}}}}}}}_{j}\right)$ is a Pearson correlation coefficient between Network_i_ and Network_j_, and *σ* is the median of *d*_*ij*_ across all possible pairs of functional networks. Then, we applied normalized-cut-based spectral clustering^123^ to group the 50 × *K* functional networks into *K* clusters. For each cluster, the functional network with the highest overall similarity with all other networks in the same cluster was selected as the most representative. The final group network atlas was composed of these maximally representative network estimations at each of the 29 resolutions studied.

In the final step, we derived each individual’s specific network atlas using NMF, initializing each participant-specific solution on the group-consensus atlas for any given scale and optimizing NMF in accordance with each individual’s specific fMRI timeseries (a 555 × 17,734 matrix). See Li et al., (2017) for further optimization detail. This procedure yielded loading matrix *V*_*i*_ (*K* × 17,734 matrix) for each participant, where each row is a functional network, each column is a vertex, and the value in each cell quantifies the extent to which each vertex belongs to each network. This probabilistic (soft) definition was converted into discrete (hard) network definitions for the display and calculation of network statistics by labeling each vertex in accordance with its highest loading. This procedure was repeated for all 29 network resolutions.

### Quantification and statistical analysis

#### Calculation of variability and spatial alignments of personalized networks

To quantify the degree to which NMF captured individualized functional neuroanatomy regardless of the NMF parameters chosen, we created individualized networks across a range of NMF parameters at both a coarse (K = 4) and fine (K = 20) scale (locality = 5, 10, 20, sparsity = 0.5, 1, and 2). After recalculating individualized networks for the 8 new parameter pairings at both scales, we calculated Adjusted Rand Indices (ARI) to evaluate the correspondence between networks derived from distinct parameterizations and our original individualized functional networks (set at spatial regularization = 10, sparsity = 1). This step yielded a distribution of within-subject ARI, or the similarity in individualized network decompositions across parameterizations. To evaluate the degree to which individual variability in functional network decompositions was driven by individual variability in the functional imaging data rather than the NMF parameters chosen, we compared the distributions of within-subject ARI to between-subject ARI across parameters. Within and between-subject ARI were calculated between our original individualized functional networks and the 16 new conditions for K = 4 and K = 20, locality/sparsity = 5 and 0.5, 5 and 1, 5 and 2, 10 and 0.5, 10 and 2, 20 and 0.5, 20 and 1, 20 and 2.

In order to quantify cross-subject spatial variability in personalized networks, we calculated the median absolute deviation (MAD) of personal network loadings at each vertex across participants. MAD is a non-parametric measure of variance that does not assume a normal distribution. First, we calculated MAD for each network at each scale. Next, MAD was averaged across *K* networks to obtain a single value of MAD at each vertex for any given scale *K*.

##### Functional hierarchy

In order to quantify networks in terms of their position within a functional hierarchy, we used a widely adopted principal gradient of functional connectivity^17^ (https://github.com/NeuroanatomyAndConnectivity/gradient_analysis). The principal gradient is derived from the primary component of variance in patterns of whole-brain functional connectivity, aligns with hierarchical estimations derived from tract-tracing^7^, and reflects a unimodal-to-transmodal continuum of cortical function^17^. As such, at each cortical vertex, the value of this gradient reflects the loading of that vertex onto a cortical hierarchy, with higher principal gradient values corresponding to higher positioning within the hierarchy.

To maximize equivalence with prior studies, we used the original map of the principal gradient provided by Margulies et al. (2016). This map was transformed to *fsaverage5* space using metric-resample from Connectome Workbench. Functional hierarchy values for each network were quantified as the average principal gradient value of each vertex within each network in group-consensus space. These network-wise hierarchy values were used to analyze the spatial distribution of the effects of age and executive function, as described below.

##### Reference networks

To allow for comparison with previously estimated cortical systems, we quantified the overlap of each group-consensus network with a commonly used 7 and 17-functional network parcellation^84^. To illustrate this overlap, we assigned colors to the group and individualized networks in accordance with their maximum overlap with networks from the 7 and 17-network parcellations.

##### Spatial permutation testing (spin test)

In order to evaluate the significance of the localization of between-participant variability (MAD) to transmodal cortical areas, we used a spatial permutation procedure called the spin test^48,117,120,124^ (https://github.com/spin-test/spin-test). The spin test is a spatial permutation method based on angular permutations of spherical projections at the cortical surface. Critically, the spin test preserves the spatial covariance structure of the data, providing a more conservative and realistic null distribution than randomly shuffling locations. Due to varying spatial covariance structures across scales, we conducted separate spin tests at each scale.

##### Modeling the association of scale with MAD-principal gradient colocalization

To account for potential non-independence of MAD-principal gradient correlations across scales, significance testing was performed using non-parametric bootstrap resampling. Specifically, we recalculated MAD and the subsequent spatial correlation with the principal gradient at each scale across 1000 bootstrap resamples to generate a bootstrapped confidence interval of the second-order relationship between the network scale and the MAD-principal gradient correlations.

#### Quantification of between-network coupling

We used functional connectivity (FC) to quantify inter-regional coupling in the processed BOLD signals. Specifically, we calculated between-network FC at three levels of analysis: network, edge, and vertex. At all levels, FC was quantified as the Pearson correlation between BOLD timeseries. At the network level, between-network connectivity was quantified as a network’s mean correlation with all other networks. At the edge level, between-network connectivity was quantified as the mean vertex-by-vertex correlation between vertices in both networks. At the vertex level, we evaluated each vertex’s average correlation to vertices from all other networks. Between-network coupling at each level was quantified separately at each scale for each participant.

#### Developmental analyses

##### Developmental modeling

Developmental effects were estimated using generalized additive models^125,126^ (GAMs) with penalized splines in R (Version 3.6.3) using the *mgcv* package^127,128^. To avoid over-fitting, nonlinearity was penalized using restricted maximum likelihood (REML). Participant sex and in-scanner head motion were included as covariates within each GAM. Head motion was quantified as the mean framewise root-mean-square displacement across the three functional runs for each subject. Age was modeled using a penalized thin-plate regression spline; covariates were modeled as parametric regressors. This model can summarized using the formula in Eq. 6:

6 $${FC} \sim s\left({{{{{{\mathrm{age}}}}}}}\right)+{{{{{{\rm{\beta }}}}}}}_{{{{{{{\mathrm{sex}}}}}}}}+{{{{{{\rm{\beta }}}}}}}_{{{{{{{\mathrm{head}}}}}}}\,{{{{{{\mathrm{motion}}}}}}}}$$

To quantify the effect sizes of each age spline, we calculated the change in adjusted *R*^2^ (Δ*R*^2^_*adj*._) between the full model and a nested model that did not include an effect of age. Statistical significance was assessed using analysis of variance (ANOVA) to compare the full and nested models. Because Δ*R*^2^_*adj*._ describes effect size but not direction (i.e., increasing or decreasing FC with age), we extracted and applied the sign of the age coefficient from an equivalent linear model as in prior work^40^. To estimate windows of significant age-related change for each network-level model, we calculated the age range for which the 95% confidence interval of estimated age splines did not include 0^129,130^. To calculate the intervals, we used the *gratia* package in R^131^. Multiple comparisons were controlled for with the false discovery rate (FDR) correction (*q* < .05).

##### Modeling the distribution of developmental effects across the functional hierarchy

After analyzing the effect of age on between-network FC, we sought to evaluate the spatial distribution of age effects along the principal gradient. At the network level, we extracted the mean hierarchy value for each network at each scale and regressed these values on the corresponding pattern of age effects (Eq. 7).

7 $${{{{{{\mathrm{Age}}}}}}}\,{{{{{{\mathrm{effect}}}}}}}({\triangle R}_{{adj}.}^{2}) \sim {{{{{{\rm{\beta }}}}}}}_{{{{{{{\mathrm{hierarchy}}}}}}}}$$

To account for potential non-independence of age effects across scales, significance testing was performed using non-parametric bootstrap resampling. Specifically, we recalculated the age effects for each network and the resulting transmodality relationship across 1000 bootstrap resamples to generate a bootstrapped confidence interval. The effect size of the second-order model was also described as a Spearman’s correlation coefficient.

We next evaluated how the magnitude of the age effects corresponded to the span of each edge (between-network connection) across the functional hierarchy. We modeled this effect in two ways. First, we calculated the difference in the hierarchy values for each pair of networks at each scale (“hierarchical distance”) and regressed this difference on the age effects from the edge-wise developmental models (Eq. 8).

8 $${{{{{{\mathrm{Age}}}}}}}\,{{{{{{\mathrm{effect}}}}}}}({\triangle R}_{{adj}.}^{2}) \sim {{{{{{\rm{\beta }}}}}}}_{{{{{{{\mathrm{hierarchical}}}}}}}\,{{{{{{\mathrm{distance}}}}}}}}$$

As above, significance was evaluated using non-parametric bootstrap resampling. As a sensitivity analysis, we repeated this procedure using the average Euclidean distance between vertices in the two networks comprising each edge. Second, we sought to visualize the interaction between hierarchical distance and age-related changes in coupling across network edges spanning different portions of the functional hierarchy. In order to continuously model the relationship between age-related changes in coupling and hierarchical distance across the functional hierarchy, we fit a bivariate smooth interaction. Specifically, we modeled the effect of transmodality on the edge-level age effects using a tensor product smooth^132^ as in Eq. 9.

9 $${{{{{{\mathrm{Age}}}}}}}\,{{{{{{\mathrm{effect}}}}}}}\left(\Delta {R}^{2}{adj}.\right) \sim {te}({{{{{{{\mathrm{Hierarchy}}}}}}}}_{{{{{{{\mathrm{Network}}}}}}}A},{{{{{{\mathrm{Hierarchy}}}}}}}_{{{{{{{\mathrm{Network}}}}}}}B})$$

To verify the statistical significance of this model, we performed the same non-parametric bootstrap procedure as above using a simplified linear interaction model.

##### Modeling scale-dependent developmental effects

In order to quantify and localize the scale-dependence of developmental changes in between-network coupling, we modeled the role of scale on coupling at each vertex. Model formulas and initial model fits were estimated using GAMs (Eq. 10).

10 $${{{{{{\mathrm{Network}}}}}}}\,{{{{{{\mathrm{coupling}}}}}}} \sim s\left({{{{{{\mathrm{Scale}}}}}}}\right)+{{{{{{\rm{\beta }}}}}}}_{{{{{{{\mathrm{sex}}}}}}}}+{{{{{{\rm{\beta }}}}}}}_{{{{{{{\mathrm{head}}}}}}}\,{{{{{{\mathrm{motion}}}}}}}}$$

GAM-derived coefficient estimates for scale, sex, and head motion were used to initialize generalized estimating equations (GEEs). GEEs enabled us to account for the covariance between same-subject measurements across scales without assuming the independence of these observations. At each vertex, the effect of the scale was assessed for statistical significance via a joint Wald test that compared the full model to a nested model that did not include an effect of scale.

Age-by-scale interactions were modeled using the same procedure. First, GAMs were used to generate initial model fits. Age-by-scale interactions were modeled as a bivariate tensor product interaction (*ti* in mgcv) as in Eq. 11.

11 $${{{{{{\mathrm{Network}}}}}}}\,{{{{{{\mathrm{coupling}}}}}}} \sim s\left({{{{{{\mathrm{Scale}}}}}}}\right)+s\left({{{{{{\mathrm{Age}}}}}}}\right)+{ti}({{{{{{\mathrm{Scale}}}}}}},{{{{{{\mathrm{Age}}}}}}})+{{{{{{\rm{\beta }}}}}}}_{{{{{{{\mathrm{sex}}}}}}}}+{{{{{{\rm{\beta }}}}}}}_{{{{{{{\mathrm{head}}}}}}}\,{{{{{{\mathrm{motion}}}}}}}}$$

Again, GEEs were used to account for the covariance between same-subject measurements across scales without assuming independence. Statistical significance was evaluated with a joint Wald test that compared the full model to a nested model that did not include a bivariate interaction term.

Finally, to further understand scale-dependent age effects within areas exhibiting age-by-scale interactions, we compared network-level developmental effects across scales for networks that fall at opposite ends of the principal axis. We grouped networks by their maximum overlap with the higher-resolution reference atlas (the 17 network solution provided by Yeo et al.) and calculated average transmodality values for each group of reference networks. The lowest (Somatomotor-A) and highest (Default mode-B) transmodality networks were chosen to depict differential scale dependence across the principal gradient. To illustrate the effect of scale, we fit a penalized spline to the relationship between scale and observed age effects for each network within each group.

### Analyses of executive function

#### Cognitive assessment

The Penn computerized neurocognitive battery (Penn CNB) was administered to all participants as part of a session separate from neuroimaging. The CNB consists of 14 tests adapted from tasks applied in functional neuroimaging to evaluate a broad range of cognitive domains^133^. These domains include executive function (abstraction and mental flexibility, attention, working memory), episodic memory (verbal, facial, spatial), complex cognition (verbal reasoning, nonverbal reasoning, spatial processing), social cognition (emotion identification, emotion differentiation, age differentiation), and sensorimotor and motor speed. Accuracy for each test was z transformed. As previously described in detail, factor analysis was used to summarize these accuracy scores into three factors^134^, including executive and complex cognition, episodic memory, and social cognition. Here, we focused on the executive and complex cognition factor score; episodic memory and social cognition factor scores were evaluated in specificity analyses.

##### Cognitive modeling

Analyses of associations with cognition were executed using GAMs, as described above for developmental analyses. Specifically, EF was modeled using a penalized regression spline, while co-varying for age using a penalized regression spline; participant sex and mean head motion were included as linear covariates (Eq. 12).

12 $${FC} \sim s\left({EF}\right)+s\left({{{{{{\mathrm{age}}}}}}}\right)+{{{{{{\rm{\beta }}}}}}}_{{{{{{{\mathrm{sex}}}}}}}}+{{{{{{\rm{\beta }}}}}}}_{{{{{{{\mathrm{head}}}}}}}\,{{{{{{\mathrm{motion}}}}}}}}$$

As for developmental analyses, we calculated the effect size as the change in adjusted *R*^2^ between the full model and a nested model that did not include the effect of EF (Δ*R*^2^_*adj*._).

##### Linking associations with EF to the principal gradient of brain organization

After analyzing the effect of cognition on between-network FC, we sought to evaluate the distribution of EF effects across the sensorimotor to association hierarchy. At the network level, we extracted the mean hierarchy value for each network at each scale and compared these values to the corresponding pattern of associations between between-network coupling and EF. As for previous developmental analyses, in order to assess the statistical significance of EF effect-hierarchy correspondence, we also evaluated a second-order model over 1000 bootstrap resamples. However, here we also included quadratic terms (Eq. 13).

13 $${EF}\,{{{{{{\mathrm{Effect}}}}}}}\left(\Delta {R}^{2}{adj}.\right) \sim {{{{{{\rm{\beta }}}}}}}_{{{{{{{\mathrm{Hierarchy}}}}}}}}+{{{{{{\rm{\beta }}}}}}}_{{{{{{{{\mathrm{Hierarchy}}}}}}}}^{2}}$$

The resulting bootstrapped confidence intervals for *β*_*Hierarchy*_ and ${{\beta }}_{{{Hierarchy}}^{2}}$ were then used for significance testing of these second-order effects.

##### Modeling scale-dependent cognitive effects

In order to quantify and localize the scale dependence of associations between EF and between-network coupling, we modeled the role of scale at each vertex. EF-by-scale interactions were modeled using the same procedure as for developmental models. First, GAMs were used to generate initial model fits. EF-by-scale interactions were modeled as a bivariate tensor product interaction (*ti* in mgcv) as in Eq. 14.

14 $${FC} \sim s\left({EF}\right)+s\left({{{{{{\mathrm{Scale}}}}}}}\right)+s\left({{{{{{\mathrm{Age}}}}}}}\right)+{ti}\left({{{{{{\mathrm{Scale}}}}}}},{EF}\right)+{ti}({{{{{{\mathrm{Scale}}}}}}},{{{{{{\mathrm{Age}}}}}}})+{{{{{{\rm{\beta }}}}}}}_{{{{{{{\mathrm{sex}}}}}}}}+{{{{{{\rm{\beta }}}}}}}_{{{{{{{\mathrm{headmotion}}}}}}}}$$

Again, GEEs were used to account for the covariance between same-subject measurements across scales without assuming independence. Statistical significance was evaluated with a joint Wald test that compared the full model to a nested model that did not include a bivariate interaction term.

Finally, to further understand scale-dependent cognitive effects within areas exhibiting EF-by-scale interactions, we compared network-level cognitive effects across scales for networks that fall at opposite ends of the functional hierarchy. To model the effect of scale, we fit a penalized spline to the relationship between scale and observed cognition effects for the lowest (Somatomotor-A) and highest (Default mode-B) order networks.

##### Multivariate EF predictions

As a final step, we sought to assess the degree to which multivariate patterns of functional edge coupling across scales jointly explain individual differences in executive function. To do this, we used ridge regression^135^. We iteratively fit a regression model to two-thirds of our sample (462 participants) and predicted executive function scores from functional coupling data in the left-out testing third of participants (231 participants). In each iteration, we used nested parameter optimization. Specifically, coefficients for each edge were fit with the 1st third of the sample, and then the L2 penalty term was selected based on predictions in the 2nd third of the sample. Finally, the degree to which functional coupling explains EF was calculated using the unseen 3rd third of the sample. In that left-out data that was not used in model training, we calculated the correlation between actual and predicted EF, as well as the mean squared error (MSE). We repeated this process 100 times to ensure that specific sample splits were not driving results, and averaged predictions across iterations. To evaluate the statistical significance of these predictions, we used permutation testing. Specifically, we repeated this process 1000 times, and compared our outcome measure (correlation of predicted vs. actual EF) versus the distribution of models where EF scores had been permuted across participants.

### Reporting summary

Further information on research design is available in the Nature Research Reporting Summary linked to this article.

## Supplementary information

Supplementary Information

Reporting Summary

## Data Availability

The Source data generated in this study have been deposited in the Zenodo database under accession 10.5281/zenodo.6288879. The raw neuroimaging data are protected and are not available due to data privacy laws.

## Code availability

The PNC data are publicly available in the Database of Genotypes and Phenotypes: accession number: phs00607.v3.p2; https://www.ncbi.nlm.nih.gov/projects/gap/cgi-bin/study.cgi?study_id=phs000607.v3.p2. All analysis code is available here https://github.com/PennLINC/multiscale, with detailed explanation provided at https://pennlinc.github.io/multiscale/.
