# Supplementary material for: Dissociable multi-scale patterns of development in personalized brain networks
Source: Nat Commun. 2022 May 12;13:2647. doi: 10.1038/s41467-022-30244-4 (PMC9098559; doi:10.1038/s41467-022-30244-4)
Supplement: Supplementary file 1 — Supplementary Information [file 41467_2022_30244_MOESM1_ESM.docx]

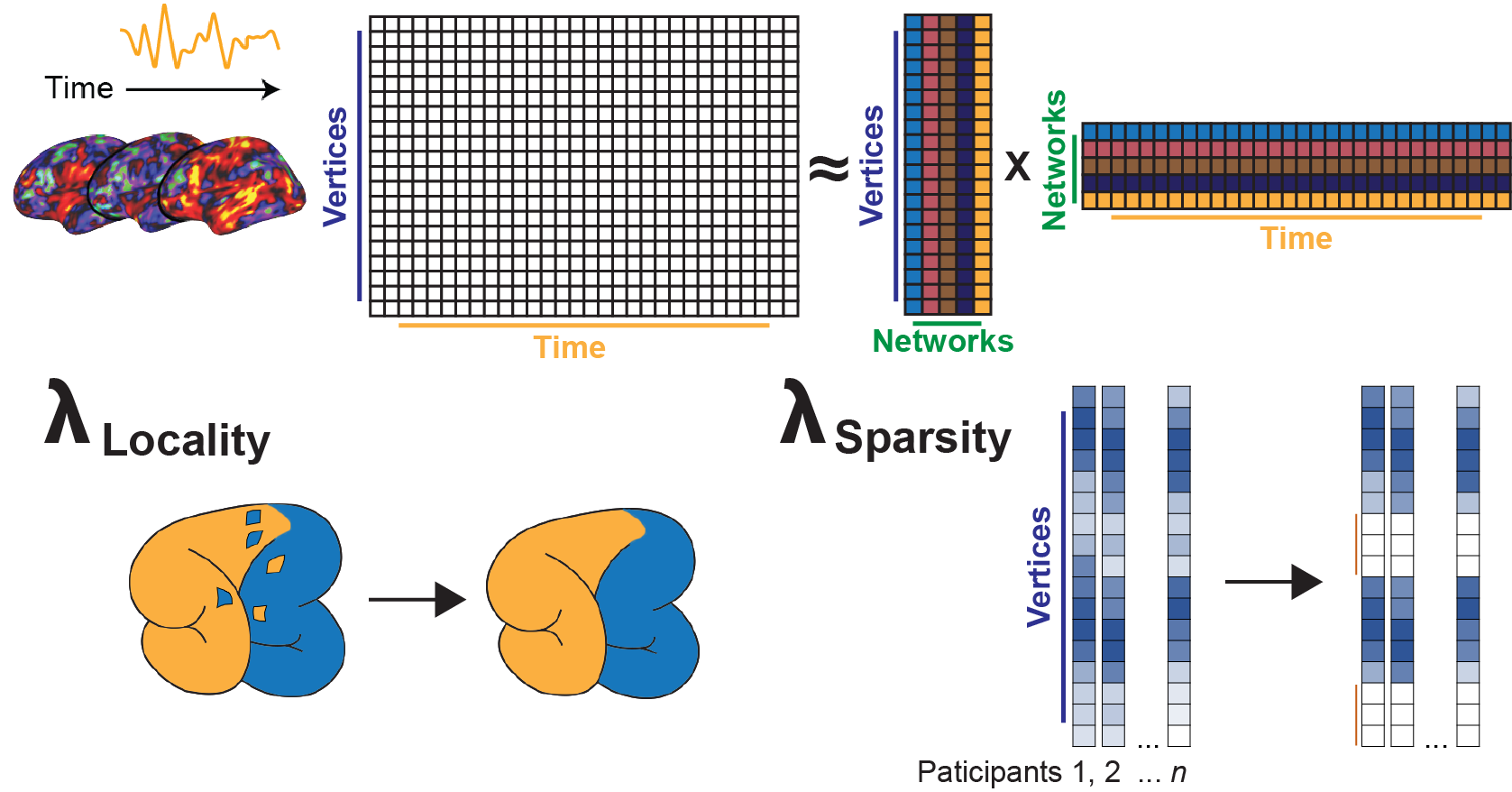


**Figure S1**: **Non-negative matrix factorization (NMF) for functional brain networks.** Overview of the NMF procedure. NMF leverages non-negative data, depicted here as values at each vertex over a functional time series, and performs matrix decompositions specialized for functional brain networks. Specifically, each matrix of vertex-level values is decomposed into two matrices: one representing latent functional network loadings across vertices, and the other representing loadings across time. Primarily, the cost function of NMF is reconstruction error: functional network distributions that minimize reconstruction error are preferred. In addition to reconstruction error, penalty terms encouraging spatial locality (λ_Locality_) and groupwise spatial sparsity (λ_Sparsity_) are enforced.


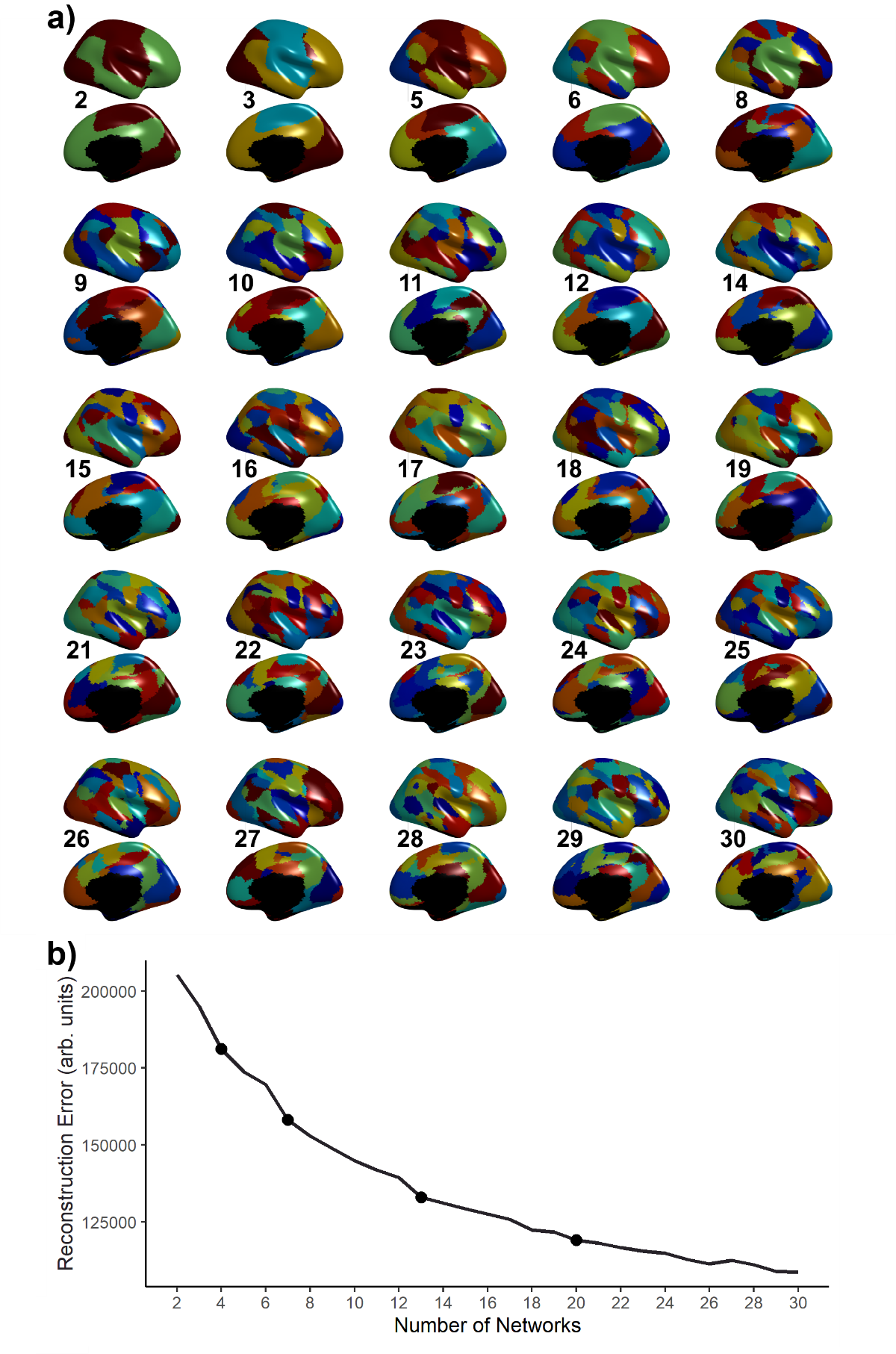


**Figure S2: Group consensus functional network atlases. a)** Group consensus atlases for all scales not depicted in the main text. **b)** Reconstruction error associated with each topological scale, averaged across participants. Reconstruction error descends smoothly from K=2 to K=30, suggesting that no single scale predominantly captures functional network organization. Scales chosen for visualization in the main text are demarcated with circles.


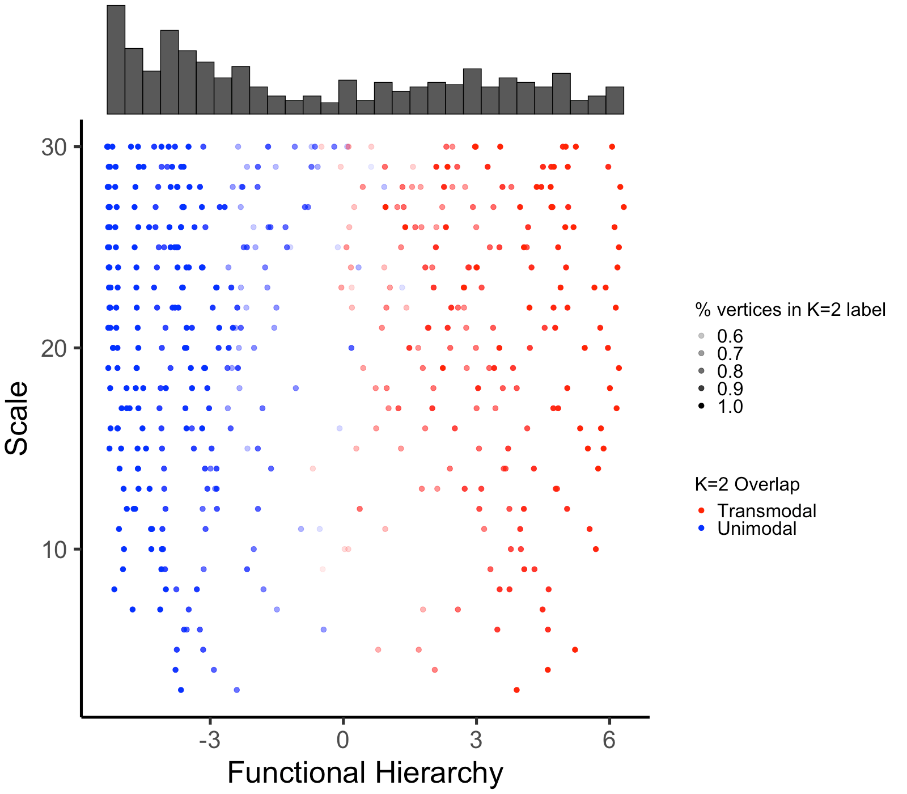


**Figure S3: Alignment of networks with a coarse unimodal or transmodal classification.** Overlap of each network with a coarse K=2 network partition, which divides the cortex into unimodal and transmodal networks. Color denotes predominant overlap while opacity denotes the percentage of vertices lying within the coarse unimodal or transmodal boundaries obtained at K=2. Across scales, 57% of finer-grained networks show more overlap with unimodal networks, whereas 43% overlapped with transmodal networks.


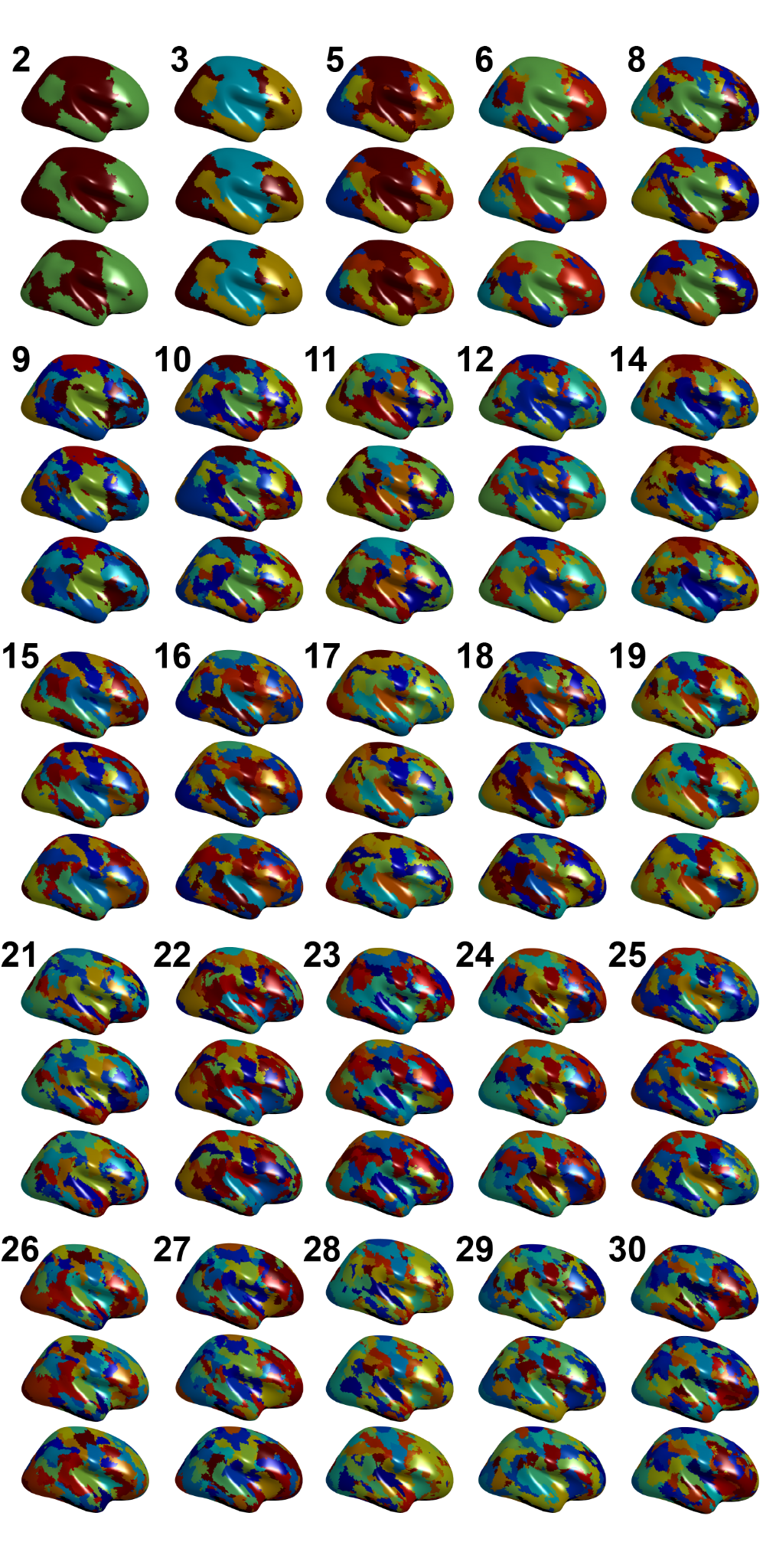


**Figure S4:** **Exemplar personalized functional networks over scales.** Personalized functional networks for all scales not displayed in the main text; the same example individuals from the main text are depicted in the same order. **
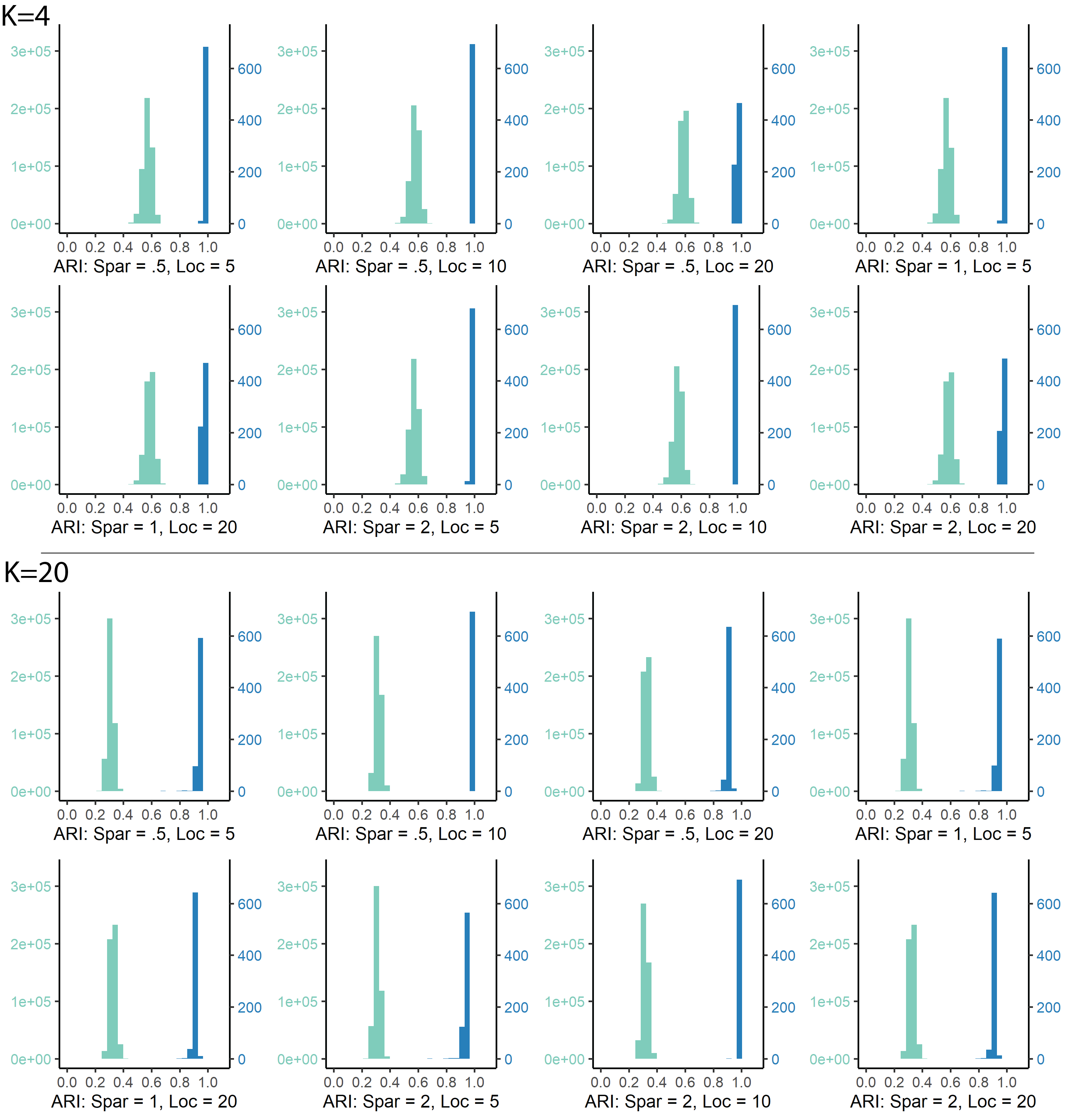
**

**Figure S5**. Same-subject networks are very similar across a range of NMF parameters. Networks derived under distinct parameterizations (locality = 5, 10, 20, sparsity = .5, 1 2) at different scales (K=4, top; K=20, bottom) are consistently more similar when derived from the same participant (blue) than different participants (teal).


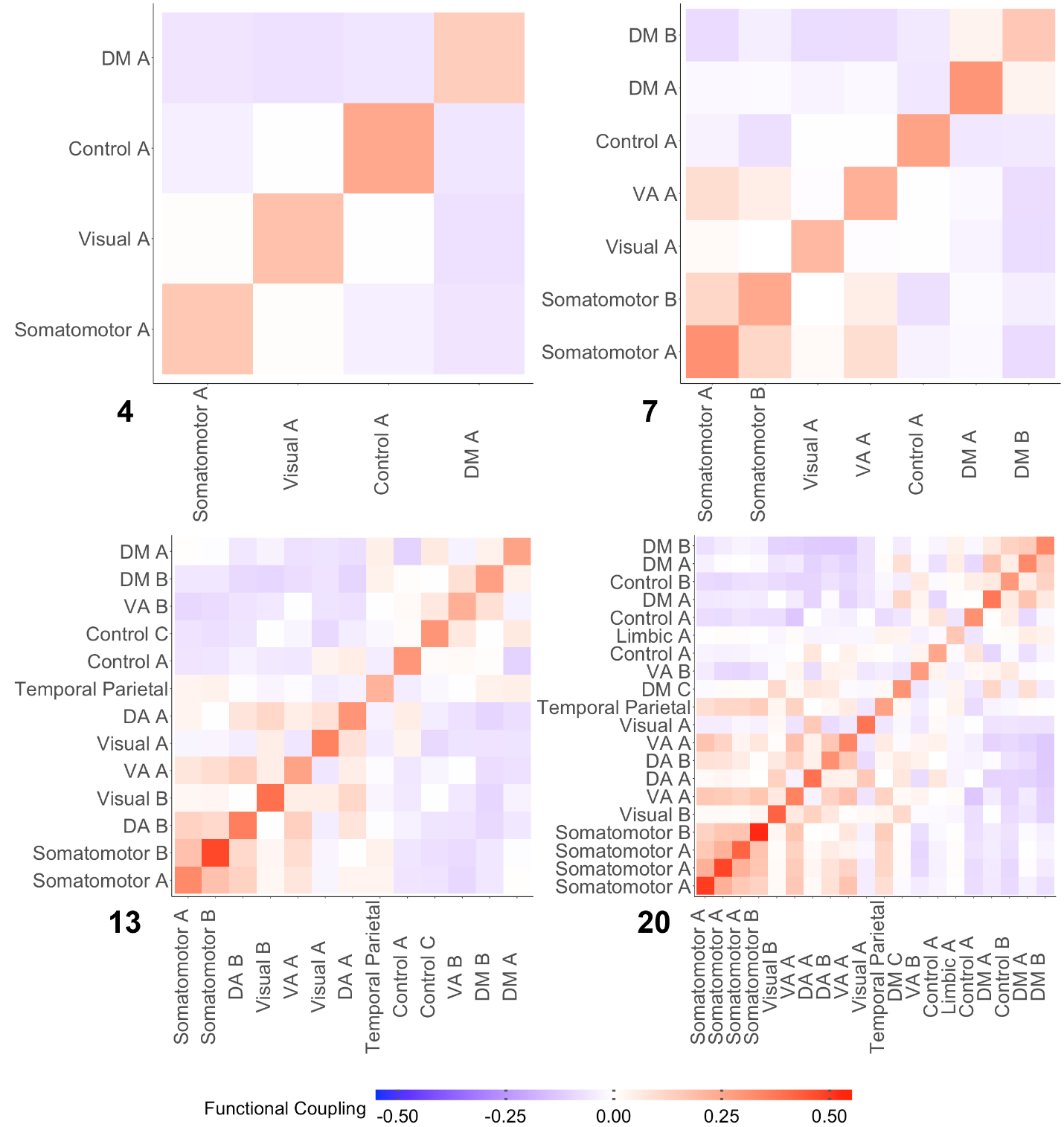


**Figure S6:** **Average functional coupling of personalized networks over example scales.** Functional connectivity matrices at the network-level at four scales. Network labels are derived from the maximal spatial overlap exhibited by each network with the 17-network solution from Yeo et al., 2011.

**
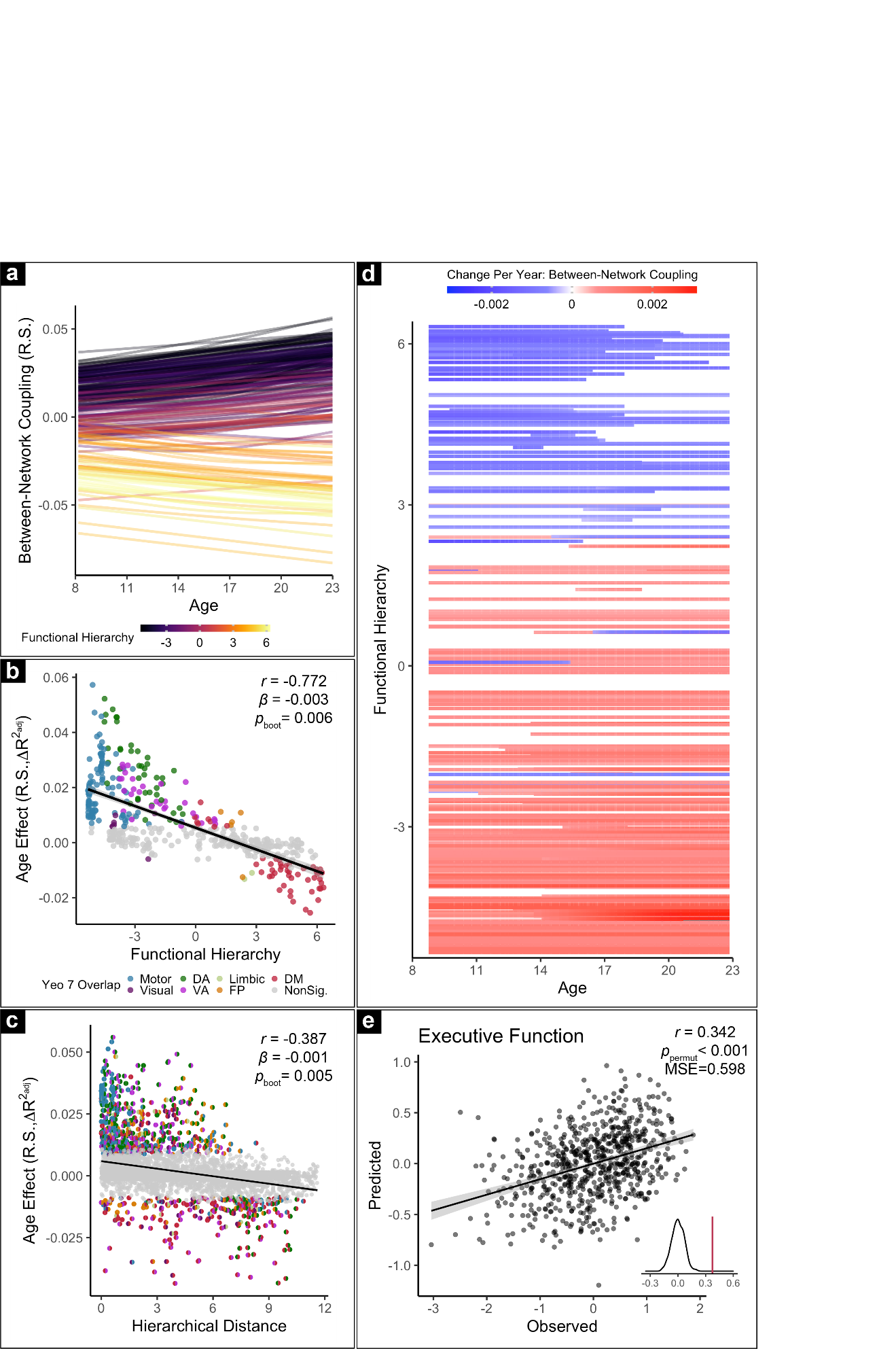
Figure S7: Sensitivity analyses using only resting-state data provide convergent results**. **a)** Between-network coupling is modeled for every network at each scale GAMs with penalized splines to account for linear and nonlinear effects of age. Each solid line represents the developmental pattern of one age-related network at one scale; colors indicate the position of that network on the functional hierarchy. Between-network coupling of sensorimotor networks increases with age, indicating increased integration. In contrast, the coupling of association networks declines with age, reflecting increased segregation. **b)** Age effects of each network are plotted versus their position on the functional hierarchy. Networks that do not display significant change over development are shaded in gray (*Q*_FDR_ > 0.05). The position of each network on the functional hierarchy explains the majority of variance in age effects (*r* = -0.772, *β* = -0.003, *p*_boot_ = 0.006). **c)** Age effects quantifying the development of between-network coupling is similarly aligned with the relative position of networks along the functional hierarchy. Age effects of every network pair at each scale are plotted versus their hierarchical distance. Network pairs without significant age effects are plotted in gray. Developmental effects on pairwise coupling between networks are associated with the hierarchical distance between networks (*r* = -0.387 *p*_boot_ = 0.005). **d)** We quantified the duration, magnitude, and direction of maturational change per year in average between-network coupling of each network across the age range studied; as in **a**, each line represents the developmental pattern of a given network at a single scale. **e)** Complex patterns of multi-scale coupling between personalized networks accurately predicts EF in unseen data. Cross-validated ridge regression with nested parameter tuning accurately predicts the EF of unseen participants using each participant’s multivariate pattern of coupling across scales. Error bands depict the 95% confidence interval. Statistical tests are two-sided, except for in **e**. MSE = mean squared error.

**
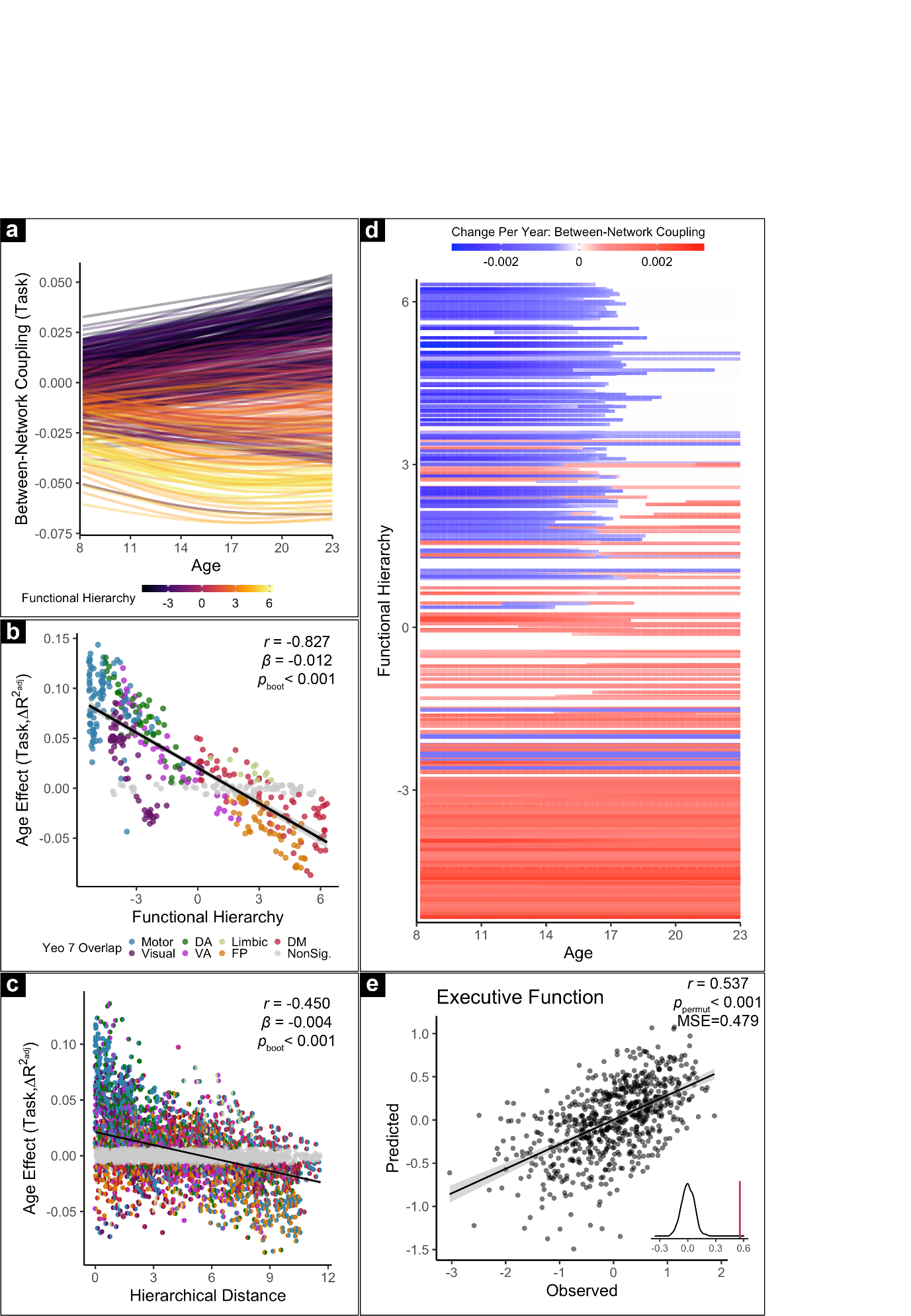
Figure S8: Sensitivity analyses using only task fMRI data provide convergent results**. **a)** Between-network coupling is modeled for every network at each scale GAMs with penalized splines to account for linear and nonlinear effects of age. Each solid line represents the developmental pattern of one age-related network at one scale; colors indicate the position of that network on the functional hierarchy. Between-network coupling of sensorimotor networks increases with age, indicating increased integration. In contrast, the coupling of association networks declines with age, reflecting increased segregation. **b)** Age effects of each network are plotted versus their position on the functional hierarchy. Networks that do not display significant change over development are shaded in gray (*Q*_FDR_ > 0.05). The position of each network on the functional hierarchy explains the majority of variance in age effects (*r* = -0.827, *β* = -0.012, *p*_boot_ < 0.001). **c)** Age effects quantifying the development of between-network coupling is similarly aligned with the relative position of networks along the functional hierarchy. Age effects of every network pair at each scale are plotted versus their hierarchical distance. Network pairs without significant age effects are plotted in gray. Developmental effects on pairwise coupling between networks are associated with the hierarchical distance between networks (*r* = -0.450 *p*_boot_ = 0.004). **d)** We quantified the duration, magnitude, and direction of maturational change per year in average between-network coupling of each network across the age range studied; as in **a**, each line represents the developmental pattern of a given network at a single scale. **e)** Complex patterns of multi-scale coupling between personalized networks accurately predicts EF in unseen data. Cross-validated ridge regression with nested parameter tuning accurately predicts the EF of unseen participants using each participant’s multivariate pattern of coupling across scales. Error bands depict the 95% confidence interval. Statistical tests are two-sided, except for in **e**. MSE = mean squared error.


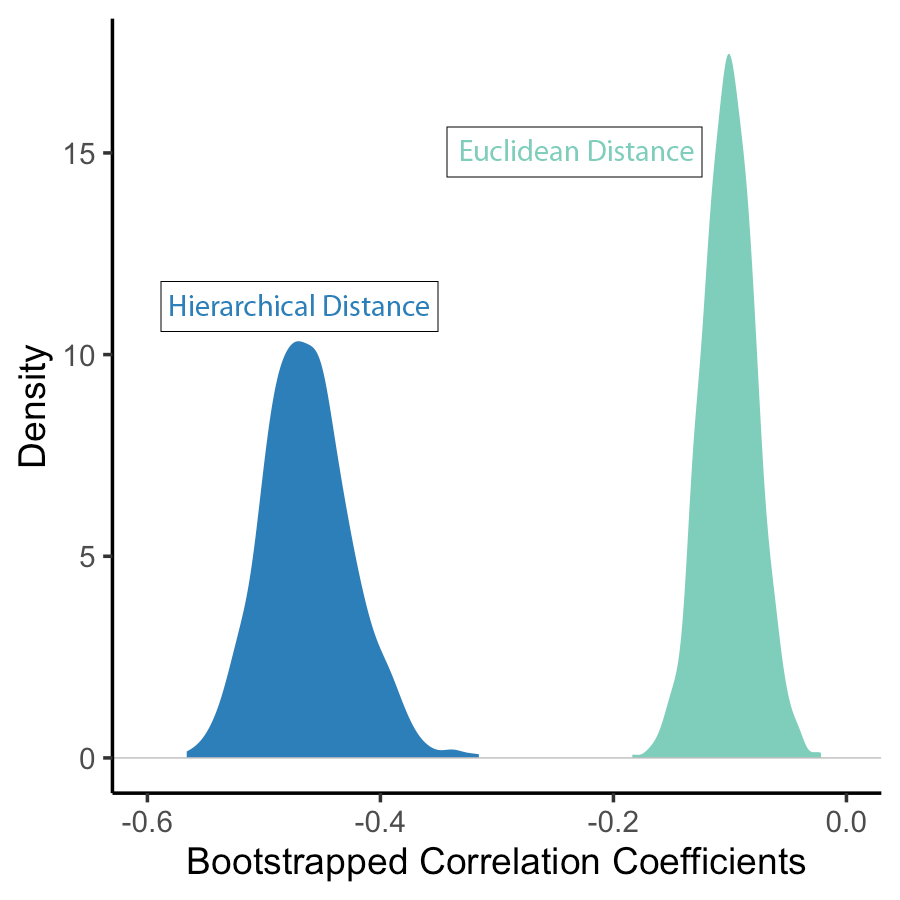


**Figure S9:** **Hierarchical distance and Euclidean distance between networks relates to developmental changes in between-network coupling.** Functional network edge development co-varied with difference in hierarchy values between networks (blue) as well as the physical distance between networks (teal). Euclidean distance is significantly negatively related to observed developmental effects, such that networks located near to each other tend to have increased coupling with age, more distant networks tend to decouple. However, these distant-dependent effects are much weaker than the effect of each network’s relative position on the sensorimotor to association axis, with hierarchical distance being more strongly related to age effects across all bootstrap resamples.


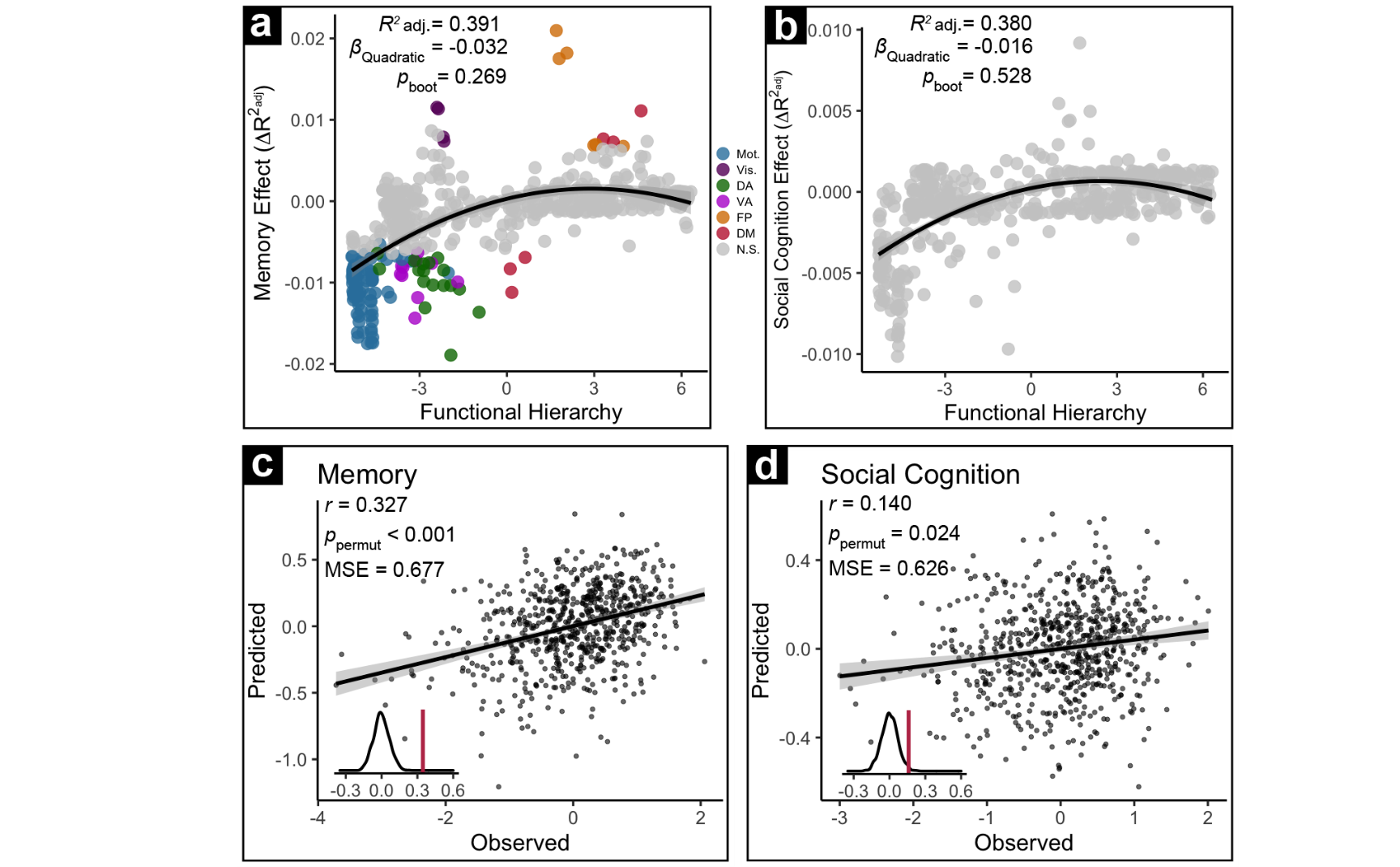


**Figure S10: Functional network coupling has limited associations with episodic memory and social cognition factor scores. a)** Network-level integration of unimodal networks was negatively associated with episodic memory; no significant associations between episodic memory and segregation of transmodal association networks were present. **b)** No significant relationships between network-level functional coupling and social cognition were present after correcting for multiple comparisons (*Q*_FDR_ > 0.05). **c)** Complex patterns of multi-scale coupling between personalized networks predicted episodic memory in unseen data, but with reduced effect size compared to EF. **d)** Complex patterns of multi-scale coupling weakly predicted social cognition in unseen participants. Statistical tests in **a** and **b** are two-sided, and are one-sided in **c** and **d**. Error bands depict the 95% confidence interval. MSE = mean squared error.
