# Supplementary material for: Dissociable multi-scale patterns of development in personalized brain networks
Source: Nat Commun. 2022 May 12;13:2647. doi: 10.1038/s41467-022-30244-4 (PMC9098559; doi:10.1038/s41467-022-30244-4)
Supplement: Supplementary file 2 — Reporting Summary [file 41467_2022_30244_MOESM2_ESM.pdf]

nature portfolio

Corresponding author(s): Theodore Satterthwaite

Last updated by author(s): Feb 28, 2022

Reporting Summary

Nature Portfolio wishes to improve the reproducibility of the work that we publish. This form provides structure for consistency and transparency in reporting. For further information on Nature Portfolio policies, see our Editorial Policies and the Editorial Policy Checklist.

Statistics

For all statistical analyses, confirm that the following items are present in the figure legend, table legend, main text, or Methods section.

n/a	Confirmed	
☐	☒	The exact sample size (*n*) for each experimental group/condition, given as a discrete number and unit of measurement
☒	☐	A statement on whether measurements were taken from distinct samples or whether the same sample was measured repeatedly
☐	☒	The statistical test(s) used AND whether they are one- or two-sided<br>*Only common tests should be described solely by name; describe more complex techniques in the Methods section.*
☐	☒	A description of all covariates tested
☐	☒	A description of any assumptions or corrections, such as tests of normality and adjustment for multiple comparisons
☐	☒	A full description of the statistical parameters including central tendency (e.g. means) or other basic estimates (e.g. regression coefficient) AND variation (e.g. standard deviation) or associated estimates of uncertainty (e.g. confidence intervals)
☐	☒	For null hypothesis testing, the test statistic (e.g. *F*, *t*, *r*) with confidence intervals, effect sizes, degrees of freedom and *P* value noted<br>*Give P values as exact values whenever suitable.*
☒	☐	For Bayesian analysis, information on the choice of priors and Markov chain Monte Carlo settings
☒	☐	For hierarchical and complex designs, identification of the appropriate level for tests and full reporting of outcomes
☐	☒	Estimates of effect sizes (e.g. Cohen's *d*, Pearson's *r*), indicating how they were calculated

Our web collection on statistics for biologists contains articles on many of the points above.

Software and code

Policy information about availability of computer code

Data collection	All MRI scans were acquired on a single 3 T Siemens TIM Trio whole-body scanner using the VB17 revision of the Siemens software. Cognitive assessments utilized the Penn computerized neurocognitive battery (Penn CNB). The CNB was administered using clickable icons on desktop or laptop computers, in a fixed order. The tests were implemented on Macintosh computers using the PowerLaboratory program (v 1.03). An Applescript routine was used to collect participant IDs and basic demographic information and to present the tests in a prescribed order.
Data analysis	All analysis code is available here https://github.com/PennLINC/multiscale, with detailed explanation provided at https://pennlinc.github.io/multiscale/.

For manuscripts utilizing custom algorithms or software that are central to the research but not yet described in published literature, software must be made available to editors and reviewers. We strongly encourage code deposition in a community repository (e.g. GitHub). See the Nature Portfolio guidelines for submitting code & software for further information.

Data

Policy information about availability of data

All manuscripts must include a data availability statement. This statement should provide the following information, where applicable:

- Accession codes, unique identifiers, or web links for publicly available datasets
- A description of any restrictions on data availability
- For clinical datasets or third party data, please ensure that the statement adheres to our policy

The PNC data is publicly available in the Database of Genotypes and Phenotypes: accession number: phs00607.v3.p2; https://www.ncbi.nlm.nih.gov/projects/gap/cgi-bin/study.cgi?study_id=phs000607.v3.p2.

Field-specific reporting

Please select the one below that is the best fit for your research. If you are not sure, read the appropriate sections before making your selection.

☒ Life sciences ☐ Behavioural & social sciences ☐ Ecological, evolutionary & environmental sciences

For a reference copy of the document with all sections, see nature.com/documents/nr-reporting-summary-flat.pdf

Life sciences study design

All studies must disclose on these points even when the disclosure is negative.

Sample size	The original Philadelphia Neurodevelopmental cohort included over 9,500 youths from the greater Philadelphia area. A subsample of these participants were invited to come to the University of Pennsylvania for neuroimaging. The neuroimaging sample (n=1,601) was designed as a large-scale developmental data resource; this sample size was not chosen based on a power analysis to detect specific effects.
Data exclusions	For inclusion in final analyses, all participants were required to have three functional runs that passed quality assurance. As in previous studies of this dataset, a functional run was excluded if mean relative root mean square (RMS) framewise displacement was higher than 0.2mm, or it had more than 20 frames with motion exceeding 0.25mm. This set of exclusion criteria resulted in a final sample of 693 participants with a mean age of 15.93 years. These criterion were preset, and have been identically applied to previous studies.
Replication	Every analysis was successfully and independently replicated by various co-authors, as detailed in our author contributions.
Randomization	There were no experimental groups. Individuals were not randomized to conditions.
Blinding	There were no experimental groups, so there was no need for blinding.

Reporting for specific materials, systems and methods

We require information from authors about some types of materials, experimental systems and methods used in many studies. Here, indicate whether each material, system or method listed is relevant to your study. If you are not sure if a list item applies to your research, read the appropriate section before selecting a response.

Materials & experimental systems

n/a	Involved in the study	
☒	☐	Antibodies
☒	☐	Eukaryotic cell lines
☒	☐	Palaeontology and archaeology
☒	☐	Animals and other organisms
☐	☒	Human research participants
☒	☐	Clinical data
☒	☐	Dual use research of concern

Methods

n/a	Involved in the study	
☒	☐	ChIP-seq
☒	☐	Flow cytometry
☐	☒	MRI-based neuroimaging

Human research participants

Policy information about studies involving human research participants

Population characteristics	After quality assurance, our final sample of 693 participants had a mean age of 15.93 years (SD = 2.33). The sample included 301 males and 392 females
Recruitment	The Philadelphia Neurodevelopmental Cohort (PNC) capitalized on the resources available through the Center for Applied Genomics at the University of Pennsylvania, including a subject pool of (at that time) approximately 50,000 genotyped youths. Critically, approximately 78% of the genotyped youths in the Center for Applied Genomic's database had provided consent to be re-contacted for future research, allowing for subjects to be approached for recruitment to the PNC. The participants were from the greater Philadelphia area and contacted after stratification by sex, age, and ethnicity. Constraints inherent to conducting safe magnetic resonance imaging could lead to a small bias in participant representation, based on exclusion criteria for MRI (e.g. implanted ferrous metal, claustrophobia, etc).
Ethics oversight	All study procedures were approved by the institutional Review Boards of both the University of Pennsylvania and the Children's Hospital of Philadelphia.

Note that full information on the approval of the study protocol must also be provided in the manuscript.

Magnetic resonance imaging

Experimental design

Design type: resting state and task fMRI

Design specifications: The n-back paradigm consisted of three conditions (0-back, 1-back, 2-back), each consisting of a 20-trial block lasting 60 seconds, and each level being repeated over three blocks. Fractals were presented for 500ms, and the inter-stimulus interval was 2,500 ms. The emotion identification paradigm consisted of 5 emotional categories in a pseudorandomized event-related design. Each emotional condition (happy, sad, anger, fear, neutral) contained 12 different faces conveying the relevant emotion, and participants were asked to decide which emotion was expressed on each face. Here, faces were displayed for 5.5 seconds followed by a variable interval (.5-18.5 seconds). Notably, our analyses aimed to capture stable functional covariance patterns rather than task-elicited. Consequently, our pipeline included a documented method for task effect regression, as detailed below.

Behavioral performance measures: Behavioral performance was evaluated out-of-scanner

Acquisition

Imaging type(s): Functional

Field strength: 3 Tesla

Sequence & imaging parameters: Prior to functional MRI acquisitions, a 5-minute magnetization-prepared, rapid acquisition gradient-echo T1-weighted (MPRAGE) image (TR = 1810 ms; TE= 3.51 ms; TI = 1100 ms, FOV = 180 x 240 mm2, matrix = 192 x 256, effective voxel resolution = 0.94 x 0.94 x 1 mm3) was acquired.

All fMRI scans were acquired with the same single-shot, interleaved multi-slice, gradient-echo, echo planar imaging (GE-EPI) sequence sensitive to BOLD contrast with the following parameters: TR = 3000 ms; TE = 32 ms; flip angle = 90; FOV = 192 x 192 mm2, matrix = 64 x 64; 46 slices; slice thickness/gap = 3/0 mm, effective voxel resolution = 3.0 x 3.0 x 3.0 mm.

A B0 field map was derived for application of distortion correction procedures, using a double-echo, gradient-recalled echo (GRE) sequence: TR = 1000ms; TE1 = 2.69ms; TE2 = 5.27ms; 44 slices; slice thickness/gap = 4/0 mm; FOV = 240mm; effective voxel resolution = 3.8 x 3.8 x 4 mm.

Area of acquisition: Whole-brain acquisitions were utilized

Diffusion MRI: ☐ Used ☒ Not used

Preprocessing

Preprocessing software: Structural images were processed with FreeSurfer (version 5.3) to allow for the projection of functional timeseries to the cortical surface. Functional images were processed using a top-performing preprocessing pipeline implemented using the eXtensible Connectivity Pipeline (XCP) Engine, which includes tools from FSL and AFNI. Co-registration of functional data to the high-resolution structural image using boundary-based registration. After projection to subject-specific FreeSurfer reconstructions, functional data was smoothed on the surface of this reconstruction with a 6-mm full-width half-maximum kernel.

Normalization: For each modality, the fMRI timeseries of each participant was projected to their own FreeSurfer surface reconstruction. This data was smoothed and projected to the template cortical surface.

Normalization template: We utilized the fsaverage5 template, which has 10,242 vertices on each hemisphere (18,715 total vertices after removing the medial wall)

Noise and artifact removal: Correction for distortions induced by magnetic field inhomogeneity utilized FSL's FUGUE utility. Despiking entailed interpolation of intensity outliers in each voxel's time series using AFNI's 3dDespike utility. Images were de-noised using a 36-parameter confound regression model that has been shown to minimize associations with motion artifact while retaining signals of interest in distinct sub-networks. This model included the six framewise estimates of motion, the mean signal extracted from eroded white matter and cerebrospinal fluid compartments, the mean signal extracted from the entire brain, the derivatives of each of these nine parameters, and quadratic terms of each of the nine parameters and their derivatives. Both the BOLD-weighted time series and the artifactual model time series were temporally filtered using a first-order Butterworth filter with a passband between 0.01 and 0.08 Hz to avoid mismatch in the temporal domain. To derive time series that were more comparable across runs, the task activation model was regressed from n-back and emotion identification fMRI data. The task activation model and nuisance matrix were regressed out using AFNI's 3dTproject. As prior, we removed vertices with low signal-to-noise ratio. We used the same SNR mask as in our prior work, which used the same dataset. After masking, 17,734 vertices remained for subsequent analyses.

Volume censoring: Outlier volumes were subject to despiking.

Statistical modeling & inference

Model type and settings	Several statistical models were used. Mass univariate analyses included vertex-wise analyses of age and executive function effects over scales using generalized estimating equations. Cross-validated ridge regression served as our multivariate method, detailed in the multivariate modeling section. Reported spatial correlations accounted for spatial autocorrelation by utilized spin-test null permutation models as well as correction for multiple comparisons.
Effect(s) tested	Effects were drawn from subject-level quantifications of between-network coupling. We tested the relationship of between-network coupling to age and executive function while controlling for variables including sex and in-scanner motion.

Specify type of analysis: ☒ Whole brain ☐ ROI-based ☐ Both

Statistic type for inference (See Eklund et al. 2016)	Analyses considered all networks or all cortical verticies.
Correction	Multiple comparisons were accounted for with FDR correction.

Models & analysis

n/a	Involved in the study
☐	☒ Functional and/or effective connectivity
☐	☒ Graph analysis
☐	☒ Multivariate modeling or predictive analysis

Functional and/or effective connectivity	Gunctional connectivity, reported as functional coupling, was leveraged throughout the work. FC was derived from Pearson correlations evaluated between regions of interest over the functional time series.
Graph analysis	Between-network coupling was evaluated at multiple levels. Network edges were quantified as the Pearson correlation between personalized networks. No edges were binarized.
Multivariate modeling and predictive analysis	Executive function was utilized as an independent variable: sensitivity analyses requested by reviewers utilized memory and social cognition scores in place of EF. Predicted vs. observed correlations (as part of out-of-sample predictions) and mean squared error are reported as evaluation metrics. Further detail is available in our methods section and on GitHub.
